# Design of Ru-Ni diatomic sites for efficient alkaline hydrogen oxidation

**DOI:** 10.1126/sciadv.abm3779

**Published:** 2022-06-01

**Authors:** Lili Han, Pengfei Ou, Wei Liu, Xiang Wang, Hsiao-Tsu Wang, Rui Zhang, Chih-Wen Pao, Xijun Liu, Way-Faung Pong, Jun Song, Zhongbin Zhuang, Michael V. Mirkin, Jun Luo, Huolin L. Xin

**Affiliations:** 1 Department of Physics and Astronomy, University of California, Irvine, Irvine, CA 92697, USA.; 2 Department of Mining and Materials Engineering, McGill University, Montreal H3A 0C5, Canada.; 3 Institute for New Energy Materials and Low-Carbon Technologies and Tianjin Key Laboratory of Photoelectric Materials and Devices, School of Materials Science and Engineering, Tianjin University of Technology, Tianjin 300384, China.; 4 Department of Chemistry and Biochemistry, Queens College–CUNY, Flushing, Queens, NY 11367, USA.; 5 Department of Physics, Tamkang University, New Taipei City 25137, Taiwan.; 6 National Synchrotron Radiation Research Center, Hsinchu 30076, Taiwan.; 7 MOE Key Laboratory of New Processing Technology for Non-Ferrous Metals and Materials, and Guangxi Key Laboratory of Processing for Non-Ferrous Metals and Featured Materials, School of Resource, Environments and Materials, Guangxi University, Nanning 530004, China.; 8 State Key Laboratory of Organic-Inorganic Composites, Beijing University of Chemical Technology, Beijing 100029, China.

## Abstract

Anion exchange membrane fuel cells are limited by the slow kinetics of alkaline hydrogen oxidation reaction (HOR). Here, we establish HOR catalytic activities of single-atom and diatomic sites as a function of *H and *OH binding energies to screen the optimal active sites for the HOR. As a result, the Ru-Ni diatomic one is identified as the best active center. Guided by the theoretical finding, we subsequently synthesize a catalyst with Ru-Ni diatomic sites supported on N-doped porous carbon, which exhibits excellent catalytic activity, CO tolerance, and stability for alkaline HOR and is also superior to single-site counterparts. In situ scanning electrochemical microscopy study validates the HOR activity resulting from the Ru-Ni diatomic sites. Furthermore, in situ x-ray absorption spectroscopy and computational studies unveil a synergistic interaction between Ru and Ni to promote the molecular H_2_ dissociation and strengthen OH adsorption at the diatomic sites, and thus enhance the kinetics of HOR.

## Teaser

Ru-Ni diatomic sites can effectively catalyze alkaline hydrogen oxidation with high activity, CO tolerance, and stability.

## INTRODUCTION

Anion exchange membrane fuel cells (AEMFCs) enabling the conversion of chemical energy directly into hydrogen energy in alkaline media have gained notable attention because of the possibility of using economical electrocatalysts, bipolar plates, air loops, and membranes (*1*, *2*). Hydrogen oxidation reaction (HOR) plays a key role in AEMFC. Under alkaline conditions, the HOR occurs according to the Tafel-Volmer or Heyrovsky-Volmer pathway: (i) Tafel step: H_2_ + 2 M* → 2 M*-H_ad_; (ii) Heyrovsky step: H_2_ + OH^−^ + M* → M*-H_ad_ + H_2_O + e^−^; (iii) Volmer step: M*-H_ad_ + OH^−^ → M* + H_2_O + e^−^, where M* denotes the surface catalytic site and H_ad_ presents the adsorbed hydrogen (*3*–*5*). Notably, HOR kinetics are quite slow in alkaline media compared with the acidic ones. For example, the HOR activity on noble metals (e.g., Pt, Pd, and Ir) drops approximately 100-fold when the electrolyte switches from acid to base (*4*, *6*–*8*). Attempting to address these limitations, several strategies have been developed for low-cost and high-performance alkaline HOR electrocatalysts, such as alloying two metals (*9*–*12*), preparing core-shell structures (*13*–*15*), engineering hybrid nanostructures (*8*, *16*, *17*), tuning crystalline structures of metals (*18*, *19*), among others (*20*). Despite the great progress mentioned above in promoting the HOR activity, it is still a great challenge to precisely design cost-effective electrocatalysts with highly efficient active sites for alkaline HOR.

Besides the well-known Pt (*6*–*8*), prior studies have confirmed that Ru (*21*, *22*), Ni (*23*, *24*), Pd (*7*), and Ir (*6*) as the catalysts exhibit good performance for alkaline HOR because of their optimal values of *H binding energy (HBE). In this study, we focused on the single-atom catalysts (SACs) and diatomic site catalysts (DASCs) constructed from these metal centers, because of their highest atom-utilization efficiency, unique electronic structures, and unsaturated coordination environments of the active centers, thus leading to superior catalytic performances (*25*–*31*).

## RESULTS

**Fig. 1.**
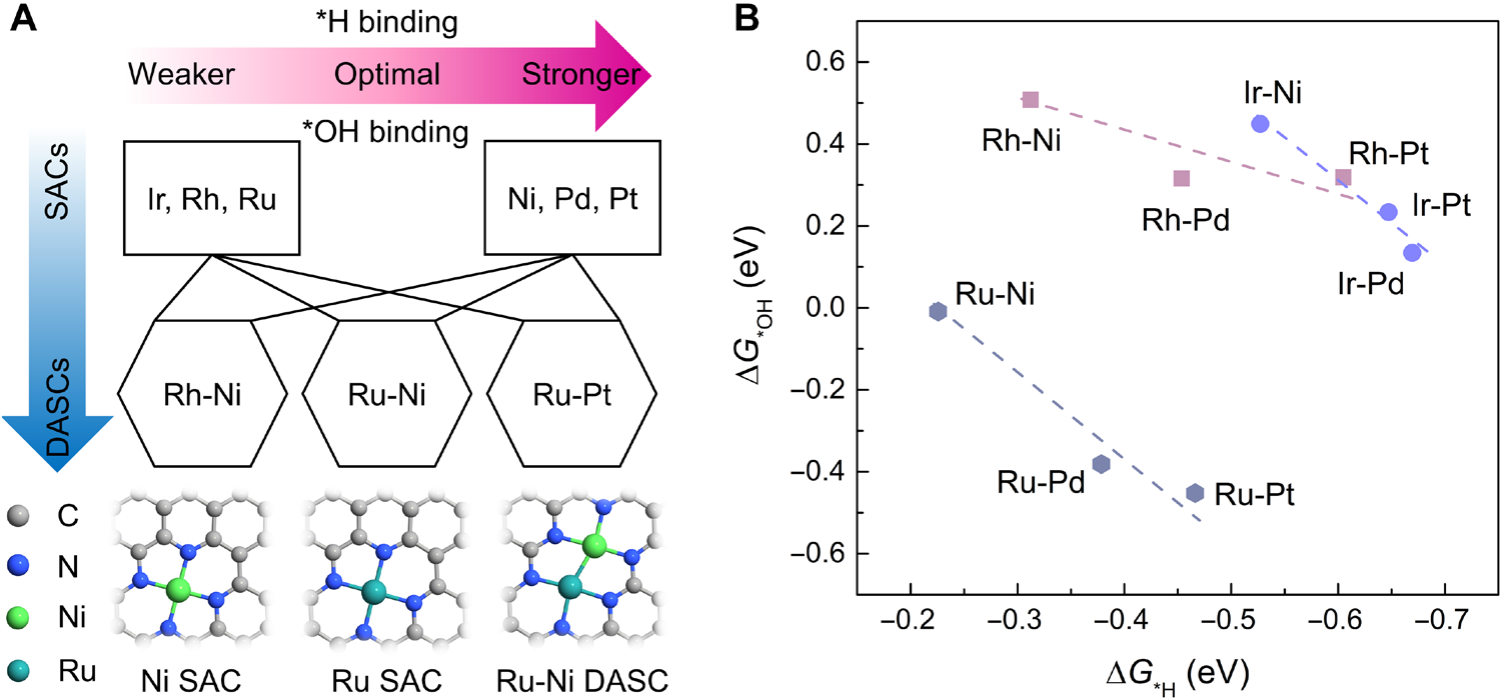
Tuning the energetics of HOR reaction intermediates by DASCs. (**A**) Tuning the binding energy of *H (ΔG_*H_) and *OH (ΔG_*OH_) via a combination of various SACs. We categorized the SACs into two different groups: strong binding—Ir, Rh, and Ru; weak binding—Ni, Pd, and Pt. The optimized structures of Ni SAC, Ru SAC, and Ru-Ni DASC are illustrated (cyan, Ru; green, Ni; blue, N; and gray, C). (**B**) ΔG_*H_ and ΔG_*OH_ of different DASCs determined by Bayesian error estimation functional with van der Waals calculations with the optimum values obtained for Ru-Ni DASC on the basis of the Sabatier principle.

We began with density functional theory (DFT) calculations not only on the HBE (ΔG_*H_) but also on *OH binding energy (OHBE, ΔG_*OH_) because the formation of an adsorbed hydroxide (*OH) was suggested to be a key factor in determining the kinetics of HOR under alkaline conditions (*32*–*34*). On pure SACs, our results revealed that the ΔG_*H_ and ΔG_*OH_ are strong on Ir, Rh, and Ru SACs, whereas they are weak on Ni, Pd, and Pt SACs (table S1). We considered combining two metal centers separately from these two groups to construct DASCs as a possible strategy to simultaneously offer active sites for the adsorption of *H and *OH with optimized values of ΔG_*H_ and ΔG_*OH_, as illustrated in Fig. 1A. For direct comparison, the SACs and DASCs were modeled as MN_4_ and M_1_N_3_M_2_N_3_ centers (M_1_ and M_2_ are metals from strong and weak categories, respectively) in a 6 × 6 graphene nanosheet to ensure a similar coordination number. According to the Sabatier principle, the binding energy of a reaction intermediate should be near an optimal value (usually around 0 eV) to allow it easily to adsorb/desorb from a certain active site and should not be too strong or too weak. Among all the considered DASCs, the computational screening suggests that Ru-Ni DASC is the best candidate for HOR with a calculated value near 0 eV for both HBE and OHBE, to promise effective and selective HOR performance (Fig. 1B). This indicates that the adsorption of *H and *OH is effectively mediated through the heterometallic bonding between Ru and Ni toward more optimal values.

**Fig. 2.**
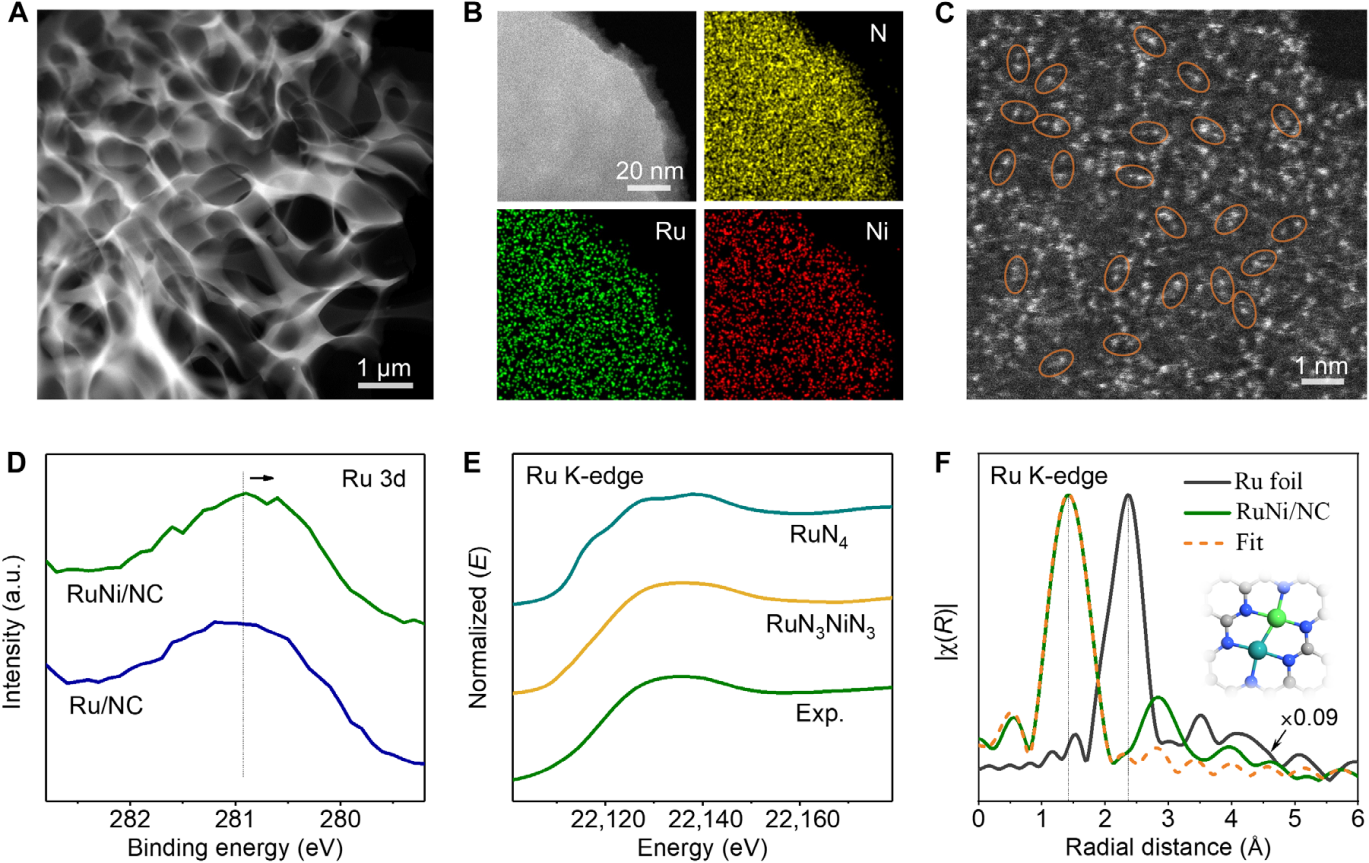
Structural characterization of RuNi/NC. (**A**) Low-magnification HAADF-STEM image. (**B**) HAADF-STEM image and corresponding STEM-EDS elemental maps of N, Ru, and Ni. (**C**) Atomic-resolution HAADF-STEM image, in which some of Ru-Ni diatomic pairs are highlighted by orange ovals. (**D**) Ru 3d XPS spectra of RuNi/NC and Ru/NC. (**E**) Comparison between the experimental Ru K-edge XANES spectrum of RuNi/NC and the theoretical ones calculated on the basis of the models of RuN_3_NiN_3_/graphene and RuN_4_/graphene, which were generated after energy optimization. (**F**) Ru K-edge FT-EXAFS spectra of Ru foil, RuNi/NC, and the fit with the RuN_3_NiN_3_/graphene model. The inset is the corresponding atomic model. a.u., arbitrary units.

On the basis of the theoretical prediction, we synthesized the Ru-Ni diatomic sites supported on N-doped porous carbon (RuNi/NC) via a dissolution-and-carbonization method (see Methods for details) (*30*). The morphology of the obtained product was characterized by the aberration-corrected high-angle annular dark-field scanning transmission electron microscopy (HAADF-STEM) and scanning electron microscopy (SEM). Figure 2A and fig. S1A show that RuNi/NC features three-dimensional (3D) interconnected carbon frameworks with randomly opened porous structures. The N_2_ adsorption-desorption isotherm and pore distribution in fig. S1 (B and C) demonstrate that RuNi/NC has a high specific area of 320.83 m^2^ g^−1^ and has many pores with sizes of 3 to 8 nm, which are advantageous for accessibility of active sites and mass transport of electrolytes during electrolysis (*28*). The x-ray diffraction (XRD) pattern in fig. S1D demonstrates that RuNi/NC is amorphous, which evidences no Ru- or Ni-related crystal particles in the sample. This result is further confirmed by elemental mapping acquired by the energy-dispersive x-ray spectroscopy (EDS; Fig. 2B), where Ru, Ni, and N homogeneously distribute over the entire architecture and no aggregation exists in the sample. Atomic-resolution HAADF-STEM images in Fig. 2C show that many spot pairs with two different contracts and an average interatomic distance of 2.4 ± 0.12 Å (fig. S1E) are distributed on the carbon matrix, suggesting the existence of abundant Ru-Ni diatomic pairs on the matrix. The Ru and Ni loadings in RuNi/NC were measured by inductively coupled plasma mass spectrometry (ICP-MS) as 5.05 and 3.57 weight % (wt %), respectively.

The x-ray photoelectron spectroscopy (XPS) and x-ray absorption fine structure (XAFS) spectroscopy were used to explore the local structure of Ru and Ni atoms in RuNi/NC. Figure 2D shows that compared with Ru/NC, Ru 3d XPS spectrum of RuNi/NC shifts toward a higher energy direction, suggesting that the introduction of Ni causes the electronic change of Ru in RuNi/NC. To further determine the coordination environment of Ru and Ni atoms in RuNi/NC, we fit the Ru and Ni K-edge x-ray absorption near edge structure (XANES) and Fourier-transformed (FT) extended XAFS (EXAFS) spectra with RuN_3_NiN_3_/graphene, RuN_4_/graphene, and NiN_4_/graphene models. As a result, the experimental Ru XANES spectrum matches better with the calculated RuN_3_NiN_3_ one than the calculated RuN_4_ one (Fig. 2E). The Ru K-edge FT-EXAFS spectrum also matches well with the fitting curve of the RuN_3_NiN_3_ model (Fig. 2F and table S2). Moreover, the Ni K-edge XANES and FT-EXAFS spectra are fitted well with the RuN_3_NiN_3_ model (fig. S2 and table S2). To explore the effect of Cl on RuN_3_NiN_3_/graphene (*31*), we built the models of Ru(Cl)N_3_NiN_3_/graphene, RuN_3_Ni(Cl)N_3_/graphene, and Ru(Cl)N_3_Ni(Cl)N_3_/graphene. We found that Cl only can be anchored to the Ru sites for Ru(Cl)N_3_NiN_3_/graphene and Ru(Cl)N_3_Ni(Cl)N_3_/graphene models, and RuN_3_Ni(Cl)N_3_/graphene is unstable. Thus, we fit the EXAFS curve of Ru K-edge in R space for RuNi/NC with Ru(Cl)N_3_NiN_3_/graphene and Ru(Cl)N_3_Ni(Cl)N_3_/graphene models. The fitting results show that the coordination numbers of Ru-Cl are close to 0 for the models (see table S2), indicating that the Cl has little effect on the RuN_3_NiN_3_ structure. Thus, we infer that the RuNi/NC sample is mainly composed of the RuN_3_NiN_3_ structure rather than the RuN_4_, NiN_4_, Ru(Cl)N_3_NiN_3_, RuN_3_Ni(Cl)N_3_, and Ru(Cl)N_3_Ni(Cl)N_3_ ones.

The control samples (Ru/NC, Ni/NC, and NC) were synthesized via the same method as that of RuNi/NC (see Methods for details). The characterizations in figs. S3 and S4 confirm that the Ru/NC and Ni/NC consist of atomically dispersed Ru and Ni sites supported on the N-doped carbon, respectively. The doped N types and contents in Ru/NC and Ni/NC are similar to those of RuNi/NC (fig. S5 and table S3), indicating that they have similar support. The Ru and Ni loadings in Ru/NC and Ni/NC are determined to be 11.37 and 8.5 wt %, respectively, by ICP-MS measurements.

**Fig. 3.**
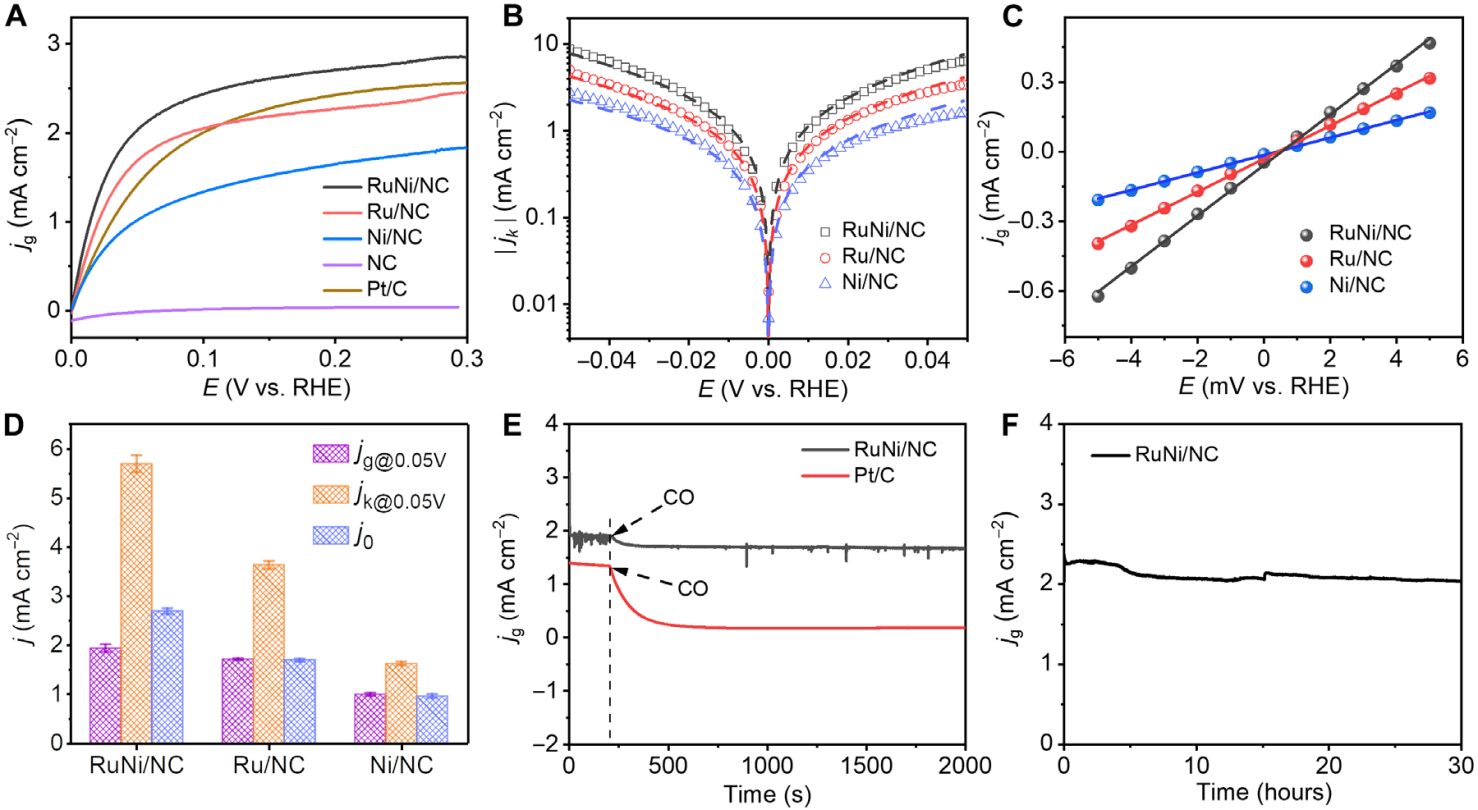
HOR performances. (**A**) Polarization curves of RuNi/NC, Ru/NC, Ni/NC, NC, and Pt/C in H_2_-saturated 0.1 M KOH at a scan rate of 1 mV s^−1^ and rotating speed of 1600 rpm. (**B**) Tafel plots of the kinetic current density on RuNi/NC, Ru/NC, and Ni/NC. The dash lines indicate the Butler-Volmer fitting. (**C**) Micropolarization region (−5 to 5 mV) of RuNi/NC, Ru/NC, and Ni/NC catalysts. (**D**) Comparison of *j*_g@0.05V_, *j*_k@0.05V_, and *j*_0_ for RuNi/NC, Ru/NC, and Ni/NC. The error bars represent the SDs of three independent measurements. (**E**) Current density–time chronoamperometry response of RuNi/NC and Pt/C in H_2_/200 ppm CO-saturated 0.1 M KOH solution at 50 mV. (**F**) Stability test of current density of Ru/NC during 30-hour HOR at 50 mV. Note that *j*_g_ represents current density normalized by the geometric area of the electrode.

The HOR performance of the RuNi/NC, Ru/NC, Ni/NC, and NC catalysts was examined in a three-electrode setup with H_2_-saturated 0.1 M KOH electrolyte. Commercial Johnson Matthey 20 wt % Pt/C was used as reference measurements. The HOR polarization curves in Fig. 3A show that the anodic current density of the RuNi/NC catalyst is higher than those of other catalysts under the whole potential range, indicating the good HOR activity of RuNi/NC. Notably, it is also superior to most of alkaline HOR catalysts (see table S4). According to the Koutecky-Levich equation (*23*), linear relationships between the inverse of overall current density at an overpotential of 50 mV versus reversible hydrogen electrode (RHE; all potentials in this work are given versus RHE unless stated otherwise) and the reciprocal of square root of the rotation rate have been plotted, which yield slopes of 4.01 cm^2^ mA^−1^ s^−1/2^ for RuNi/NC (fig. S6), close to the theoretical value of 4.87 cm^2^ mA^−1^ s^−1/2^ for the two-electron transfer of HOR (*35*).

We obtained the kinetic current density (*j*_k_) for RuNi/NC, Ru/NC, and Ni/NC according to the Koutecky-Levich equation (*23*). To understand the intrinsic HOR activity, we evaluated their mass-specific kinetics current density (*j*_m,k_) and turnover frequency (TOF). The *j*_m,k_ and TOF values were determined to be $132.6\pm 3.3\;\text{mA}\;{\text{mg}}_{\text{RuNi}}^{-1}$ and (1.07 ± 0.02) × 10^−1^ s^−1^ for RuNi/NC, $62.4\pm 2.5\;\text{mA}\;{\text{mg}}_{\text{Ru}}^{-1}$ and (0.67 ± 0.01) × 10^−1^ s^−1^ for Ru/NC, and $38.5\pm 1.3\;\text{mA}\;{\text{mg}}_{\text{Ni}}^{-1}$ and (0.23 ± 0.006) × 10^−1^ s^−1^ for Ni/NC, indicating the superior intrinsic HOR activity of RuNi/NC than Ru/NC and Ni/NC. To the best of our knowledge, the obtained *j*_m,k_ value for RuNi/NC outperforms those of the best-reported alkaline HOR catalysts (table S4). A logarithmic function of *j*_k_ versus the working potential and their Butler-Volmer fitting (*23*) are presented in Fig. 3B. By the fitting, the value of exchange current density (*j*_0_) for RuNi/NC was determined to be 2.69 ± 0.06 mA cm^−2^, higher than those for Ru/NC (1.69 ± 0.03 mA cm^−2^) and Ni/NC (0.95 ± 0.04 mA cm^−2^). By linear fitting of micropolarization regions (from −5 to 5 mV) (*11*), we also identified the exchange current density (*j*_0_). The values for RuNi/NC, Ru/NC, and Ni/NC are 2.80, 1.83, and 0.94 mA cm^−2^, respectively (Fig. 3C), which are consistent with the counterparts obtained from the Butler-Volmer fitting (Fig. 3B). These findings demonstrate that RuNi/NC has a faster HOR kinetics, compared with Ru/NC and Ni/NC (Fig. 3D).

We further examined the HOR activities of the RuNi/NC catalyst in the presence of CO. As shown in Fig. 3E, the RuNi/NC catalyst only has a small decrease of 10% over the overall current density at 50 mV during HOR electrolysis in the presence of 200 parts per million (ppm) CO, far better than the Pt/C catalyst. In addition, the cycling tests also confirm the stable HOR activity of RuNi/NC with a good CO tolerance (fig. S7), which can be attributed to easy desorption of adsorbed *CO because of the strong adsorbate-adsorbate interaction (figs. S8 and S9). Furthermore, the current density for the HOR on RuNi/NC only decreases by 11% after continuous operation for 30 hours (Fig. 3F and fig. S10), better than the counterpart of Pt/C (fig. S11). These results indicate the high electrocatalytic stability of RuNi/NC, which can be attributed to the strong interaction between the metallic atoms and the N-doped carbon support (*36*). Furthermore, the AEMFC composed respectively of RuNi/NC, Ru/NC, and Ni/NC with the same loading as an anodic catalyst, and 40 wt % Pt/C as a cathode shows a peak power density of 540 mW cm^−2^ for RuNi/NC (fig. S12A), better than those of the commercial Ru/C (~250 mW cm^−2^) (*37*) and NiMo alloy (120 mW cm^−2^) (*38*), as well as exceeding those of Ru/NC (223 mW cm^−2^) and Ni/NC (42 mW cm^−2^; fig. S12A). Moreover, the fuel cell driven by the RuNi/NC exhibits slight drop of the voltage throughout the 10-hour test at current density of 500 mA cm^−2^ (fig. S12B), making possible the application of RuNi/NC in the fuel cell.

**Fig. 4.**
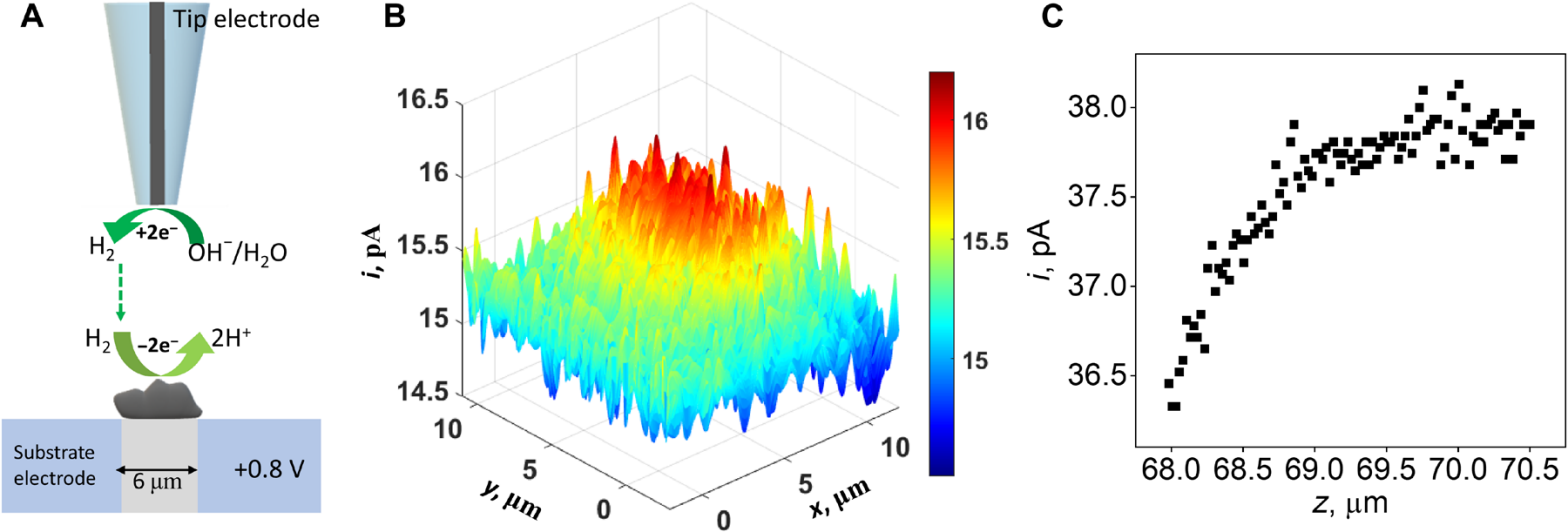
SECM measurements of HOR activity of RuNi/NC and NC catalysts in basic solution. (**A**) Schematic representation of probing electrocatalytic activity for HOR of the RuNi/NC catalyst by TG-SC mode of SECM experiments. (**B**) SECM TG-SC mode image of the RuNi/NC sample. The solution contains 0.1 M PB (pH 10). *E*_S_ = +0.8 V, *E*_T_ = −1.2 V versus Ag/AgCl. (**C**) SECM approach curve recorded at the NC electrode in 0.1 M PB (pH 10). *E*_S_ = +0.8 V, *E*_T_ = −1.2 V versus Ag/AgCl. *E*_T_ is tip potential, and *E*_S_ is substrate potential.

The catalytic activity of micrometer-sized RuNi/NC toward HOR was mapped in basic 0.1 M phosphate buffer (PB) solution at pH 10 by using a scanning electrochemical microscope (SECM) in the tip generation–substrate collection (TG-SC) mode. We used relatively large nanoelectrodes (*a* ~ 250 nm) for TG-SC mapping because micrometer-sized N-doped porous carbon sheets are relatively large, nonflat, and rough (fig. S1A, SEM image) as compared to the specimens imaged in our previous SECM studies of 2D electrocatalysts (*39*–*41*). In Fig. 4A, the tip electrode was biased at −1.2 V versus Ag/AgCl and positioned at a close distance (e.g., comparable to tip radius, *a*, determined from the SECM negative approach curve at the tip electrode; fig. S13) from the substrate (see Methods for experimental details). H_2_ generated at the tip through H_2_O/OH^−^ reduction diffused to and was oxidized at the substrate sample (biased at +0.8 V). The fast HOR at the RuNi-porous C leads to a higher substrate current (~16 pA) than the background current (~15.3 pA) in the TG-SC image, as shown in Fig. 4B. In a control experiment, the activity of the NC sample containing no RuNi was measured using the SECM substrate approach curve in TG-SC mode. When H_2_ produced at the tip electrode was not oxidized at the substrate, the substrate current decreased with the tip electrode approaching it due to the blocking of the background current. This result can be seen in Fig. 4C, pointing to the immeasurably low activity of the NC sample containing no Ru-Ni diatomic sites.

We also used SECM feedback mode to image the conductivity and HOR activity of RuNi/NC and NC catalysts in acidic solution. In this mode, either Fc/Fc^+^ (fig. S14A) or H^+^/H_2_ (fig. S14B) couple was used as the redox mediator. The tip electrode was held at a close distance from the substrate during mapping (see Methods for experimental details). The feedback mode SECM images in 1 mM Fc solution (fig. S14, C and E) were obtained with substrate unbiased. With an Fc redox mediator, the tip current was higher (positive feedback; see Methods for experimental details) above both RuNi/NC and NC than over the surrounding indium tin oxide (ITO) glass due to the fast electron transfer at its surface and high lateral conductivity, consistent with the intrinsic conductive nature of N-doped porous carbon. However, with H^+^/H_2_ used as the redox mediator (fig. S14B), the positive feedback was only observed over the diatomic RuNi catalyst (fig. S14D), while bare N-doped carbon showed negative feedback (fig. S14F), indicating that the sample activity for HOR comes from Ru-Ni diatomic sites.

**Fig. 5.**
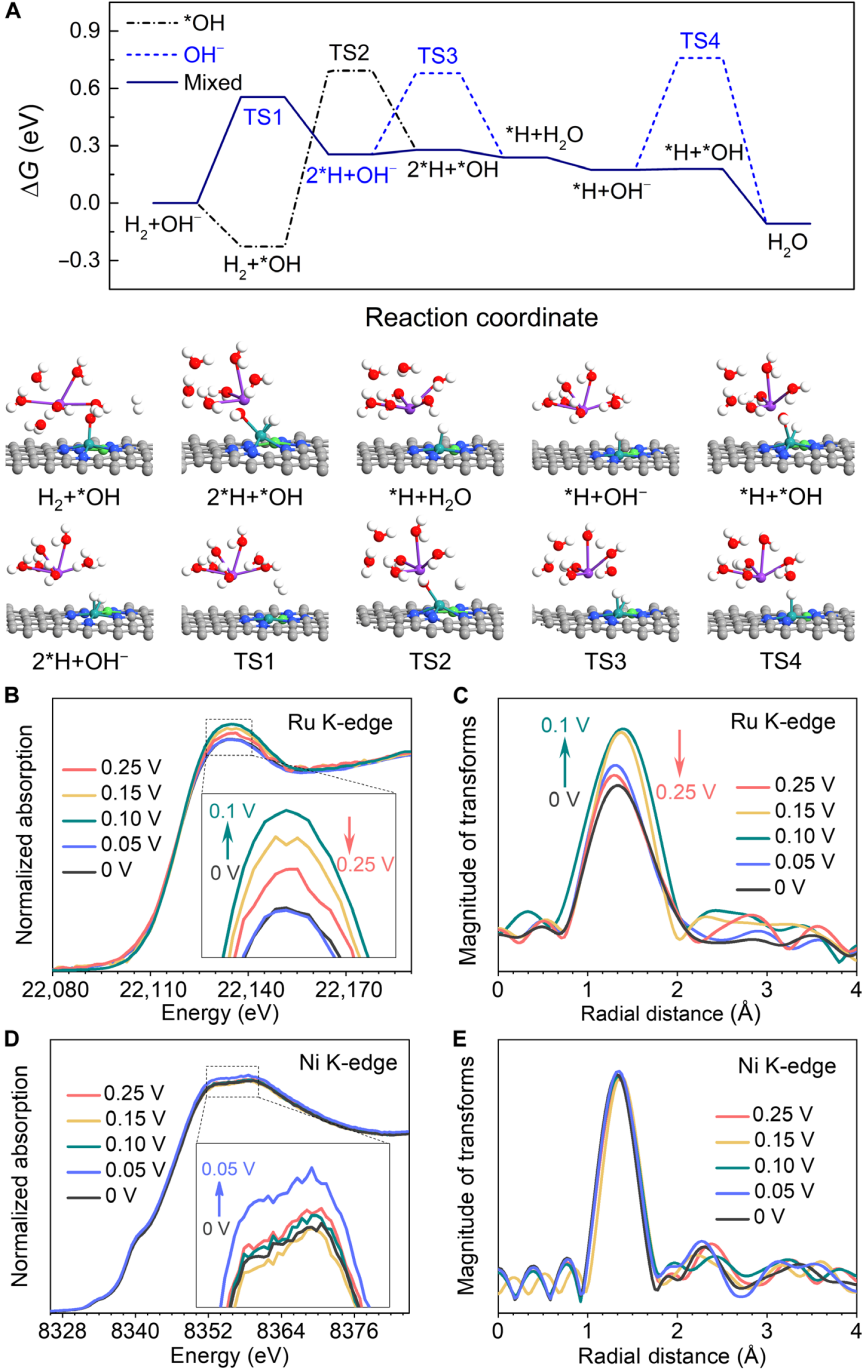
HOR mechanism on RuNi/NC. (**A**) Free energy diagram and optimized geometries of key reaction intermediates and TS in HOR on Ru-Ni DASC obtained from DFT calculations. Black and blue dashed lines represent the *OH and OH^−^ pathways, whereas navy solid line is the proposed mixed pathway. The white, gray, blue, red, purple, green, and cyan balls represent H, C, N, O, K, Ni, and Ru, respectively. (**B** to **E**) In situ XAFS measurement on RuNi/NC during HOR at different applied potentials. (B and C) Ru K-edge XANES and FT-EXAFS spectra. (D and E) Ni K-edge XANES and FT-EXAFS spectra.

To obtain a mechanistic understanding of the superior HOR performance on the RuNi/NC, we further performed DFT calculations to search for the transition state (TS) and explore the dissociation of H_2_ and the formation of H_2_O between *H and *OH/OH^−^. We considered two different sequences of OH^−^ adsorption and H_2_ dissociation, i.e., (i) OH^−^ pathway: H_2_ directly dissociates into two *H (Tafel step) and adsorb on the Ru atop site and Ru-Ni bridge site followed by the adsorbed *H combining with OH^−^ in the aqueous solution (Volmer step); (ii) *OH pathway: OH^−^ firstly adsorb on the Ru site and then H_2_ dissociates into two *H and adsorb on the Ru atop site and Ru-Ni bridge site, and then adsorbed *H and *OH combine to form H_2_O, as summarized in Fig. 5A. We postulate that the reaction mechanism is a mixed pathway, as follows: first, H_2_ dissociates into 2*H either when OH^−^ remains in the aqueous solution or adsorb on the Ru atom in Ru-Ni DASC (OH^−^ pathway is favored considering a larger Δ*G* of TS2 compared with that of TS1) and then followed by OH^−^ being adsorbed on the Ru site. The second *H prefers to combine with *OH because it is a facile process, to account for the large Δ*G* of TS4 for *H + OH^−^ to form H_2_O. Compared with the SACs (fig. S15), the Ru and Ni atoms in Ru-Ni DASCs not only provide active sites for mixed pathways of H_2_ dissociation but also enable the *OH adsorption to realize a facile H_2_O formation, which contributes significantly to the enhanced HOR performance.

We then conducted in situ XAFS measurements at the Ru and Ni K-edge of RuNi/NC during HOR in the H_2_-saturated 0.1 M KOH electrolyte to validate the proposed reaction mechanism. Figure 5B shows that the main K-edge peaks of Ru XANES spectra get higher from 0 to 0.1 V and lower from 0.1 to 0.25 V, indicating the increase in unoccupied state of the Ru 5p orbital from 0 to 0.1 V and the decrease from 0.1 to 0.25 V. The first-shell peaks of Ru K-edge FT-EXAFS spectra also have the same trend (Fig. 5C). The increase is contributed by the adsorbed *OH on the Ru top site and the decrease by the *OH desorption to release the H_2_O molecule. By contrast, the intensity of the Ni K-edge peak only increases from 0 to 0.05 V and then almost goes back to the original value when over 0.05 V (Fig. 5D), while the first-shell peaks of Ni K-edge FT-EXAFS spectra have almost no change (Fig. 5E). The increase could result from the dissociation of molecular H_2_ and adsorption on the Ni top site. These findings are consistent with the DFT calculation results in Fig. 5A and support the proposed mechanism.

## DISCUSSION

To summarize, we developed a design strategy for targeting an optimum HOR catalyst with combined theoretical and experimental efforts and deepened the understanding of the HOR mechanism by in situ techniques. Starting out, our DFT calculations predict that the Ru-Ni diatomic site is the best active center for the HOR among the screened systems of single-atom and diatomic sites. As a result, the catalyst with the Ru-Ni diatomic sites exhibits excellent HOR activity with a mass-specific kinetic current density of $132.6\pm 3.3\;\text{mA}\;{\text{mg}}_{\text{RuNi}}^{-1}$ at an overpotential of 50 mV and exchange current density of 2.69 ± 0.06 mA cm^−2^ in alkaline electrolyte, which is superior to single-site counterparts and most of alkaline HOR catalyst. The catalyst also displays excellent CO tolerance and HOR stability. SECM study reveals that the superior electrochemical activity of RuNi/NC results from the Ru-Ni diatomic sites. DFT calculations and in situ x-ray absorption spectroscopy studies further unveil a synergistic interplay between Ru and Ni in promoting molecular H_2_ dissociation and strengthening OH adsorption during the HOR.

## METHODS

### Synthesis

In a typical procedure, 144 mg of anhydrous glucose (C_6_H_12_O_6_), 6.81 mg of ruthenium (III) chloride (RuCl_3_), 3.19 mg of nickel (II) nitrate hexahydrate (H_12_N_2_NiO_12_), and 690 mg of hydroxylammonium chloride ((NH_3_OH)Cl) were ultrasonically dissolved in 80 ml of deionized water-ethanol solution (with a volume ratio of 1:1) to form a transparent solution. The solution was placed in a drying oven at 70°C for 24 hours, and the obtained solid was ground into powder, placed in a porcelain boat, heated in a tube furnace to 550°C with a rate of 5°C min^−1^, and carbonized at 550°C for 4 hours under Ar protection atmosphere. The obtained black product was ground into fine powder and denoted as RuNi/NC.

The Ru/NC was prepared with the same procedures as those of RuNi/NC except for only 9.09 mg of RuCl_3_ but no Ni precursor added. The Ni/NC was prepared with the same procedures as those of RuNi/NC except for only 12.76 mg of H_12_N_2_NiO_12_ but no Ru precursor added. The NC was prepared with the same procedures as those of RuNi/NC except for neither Ru nor Ni precursors added.

### Characterizations

XRD patterns were collected using an x-ray diffractometer (Rigaku D/max 2500) at a scan rate of 10° min^−1^ in the 2θ range of 10° to 90°. SEM observations were performed using a field emission gun (FEG) SEM instrument (Verios 460 L of FEI). Low-magnification HAADF-STEM images were recorded using an FEI Talos F200X S/TEM with a FEG. Atomic-resolution HAADF-STEM images and EDS elemental maps were taken using an FEI Titan Cubed Themis G2 300 S/TEM with a probe corrector. XPS measurements were performed using a Kratos AXIS Ultra DLD system with the Al Kα radiation as the x-ray source. N_2_ sorption/desorption measurements were performed at 77 K using an autosorb iQ instrument (Quantachrome, US) with the Brunauer-Emmett-Teller method. Pore size distribution was obtained from the N_2_ sorption/desorption isotherm. The Ru and Ni K-edge XAFS spectra were recorded at beamline TPS 44A of the National Synchrotron Radiation Research Center. Metal contents of the samples were analyzed by ICP-MS (Thermo Fisher iCAP RQ ICP-MS).

### XANES calculation

In this work, we have performed the theoretical XANES calculations to explore the local structures of Ru and Ni elements in RuNi/NC within the FDMNES package in the framework of real-space full multiple-scattering scheme with the muffin-tin approximation that is realized by applying Green function in FDMNES. The energy-dependent exchange-correlation potential was calculated in the real Hedin-Lundqvist scheme, and then the XANES spectra were convoluted using a Lorentzian function with an energy-dependent width to account for the broadening from both the core-hole and final-state widths. A cluster of 10.0-Å radius containing ~100 atoms was used in the calculation with satisfactory convergence being achieved. The structural models for XANES fitting were built on the basis of the EXAFS fitting results and were further optimized by DFT. For the simulation of Ni edge, the cutoff of the occupied states and orbit spin are considered.

### EXAFS fitting

The EXAFS fitting was performed for the FT k^2^-weighted experimental EXAFS signals using Artemis software. The EXAFS data were preprocessed and normalized in Athena software. Fitting includes both the imaginary and real parts of the FT EXAFS oscillations and minimized the difference between the normalized experimental data and the theoretical EXAFS. The multiple-scattering path for the given atomic structure model is calculated using the FEFF8-lite code in Artemis.

### Electrochemical test

All electrochemical tests were carried out on an electrochemical workstation (CHI 760E) with a standard three-electrode system under the 0.1 M KOH solution and at room temperature (25°C). A graphite rod and Hg/HgO electrode were applied as the counter electrode and reference electrode, respectively. The glassy carbon electrode loading catalyst ink was performed as the working electrode. To prepare the catalyst ink, 5 mg of the sample was dispersed in a solution containing 500 μl of 0.5 wt % Nafion and 500 μl of ethanol, and then the ink was dispersed by ultrasound for at least 30 min. After that, 10 μl of solution was dropped onto the glassy carbon electrode (0.196 cm^−2^ for active geometric area) and dried at room temperature. The catalyst loading of RuNi/NC on the glassy carbon electrode was 0.5 mg cm^−2^. As for the HOR experiments, linear sweep voltammetry (LSV) was tested with sweep rates of 1 mV s^−1^ at a rotation rate of 1600 rpm in the H_2_-saturated electrolytes. A total of 90% iR compensation was applied to all initial data except stability data. The cyclic voltammetry (CV) range is from −0.05 to 0.3 V. The CV was scanned 50 times before scanning LSV.

The kinetic current density (*j*_k_) is obtained according to the Koutecky-Levich equation (*23*): 1/*j* = 1/*j*_k_ + 1/*j*_d_ = 1/*j*_k_ + 1/(*Bc*_0_ω^1/2^), the measured current can be divided into kinetic and diffusional components; in this equation, *j* is the measured current density, *j*_k_ is the kinetic current density, *j*_d_ is the diffusion-limited current density, *B* is the Levich constant kinetic, *c*_0_ is the solubility of H_2_ in KOH electrolyte, and ω is the rotating speed.

The diffusion limiting current density (*j*_d_) is derived first by the equation *j*_d_ = 0.62*nFD*^3/2^ν^−1/6^*c*_0_ω^1/2^ = *Bc*_0_ω^1/2^ according to the literature (*16*, *23*). It depends on the rotation rate. The *j*_k_ is then calculated by the Koutecky-Levich equation (*23*): 1/*j* = 1/*j*_k_ + 1/*j*_d_ = 1/*j*_k_ + 1/(*Bc*_0_ω^1/2^) with the calculated *j*_d_.

The TOF is calculated with the equation (*42*): TOF = (*j*_k_*M*_metal_)/(*c*_cat._ω_metal_*F*), where *j*_k_ is the kinetic current density, *M*_metal_ is the molar mass of metal element, *c*_cat._ is the mass loading of catalyst on the electrode, ω_metal_ is the mass ratio of metal in catalyst, and *F* is Faraday constant (96,485 C mol^−1^).

### AEMFC assembly

Catalyst ink was obtained by mixing the powdered catalyst with 5 wt % Nafion ionomers diluted by isopropanol/water with a volume ratio of 25:1. This ink was ultrasonically treated for 3 hours and sprayed on two sides of 20-μm PAP-TP-85 membrane (an active area of 5 cm^2^). RuNi/NC, Ru/NC, and Ni/NC were used as anodic catalysts with a loading of 1 mg cm^−2^, while 40 wt % Pt/C was used as a cathodic catalyst with a loading of 0.2 mg_Pt_ cm^−2^. The catalyst-coated membrane and gas diffusion layer were assembled in a standard test cell of 5.0625-cm^2^ fixture. Then, we tested them using Scribner 850e as condition-controlled fuel cell test station. The fuel cell polarization curve was recorded at 80°C and 100% relative humidity. For hydrogen supply and oxygen supply, the back pressures were fixed at 200 kPa, and gas flow rates were fixed at 500 ml min^−1^.

### SECM electrodes and electrochemical experiments

Pt nanoelectrodes with the tip radius (*a*) ranging from 100 to 250 nm were fabricated by pulling and heat sealing 25-μm-diameter Pt wires into borosilicate glass capillaries under vacuum with a P-2000 laser pipette puller as described previously (*43*). The fabricated nanoelectrodes were polished on a 50-nm alumina pad (Precision Surfaces International) under video microscopic control. Appropriate protection was used to avoid electrostatic damage to the nanotips (*44*). The three-electrode setup was used with a commercial Ag/AgCl serving as a reference electrode and a 1-mm Pt wire as a counter electrode. N-doped porous carbon sheets were deposited on the surface of ~6-μm-diameter carbon fiber microelectrode and ITO glass in basic and acidic experimental conditions, respectively, used as the SECM substrate.

### SECM setup and procedures

SECM experiments were carried out using the previously described home-built instrument (*39*). The tip was brought within a ~30-μm vertical distance from the substrate using a manual micromanipulator under an optical microscope. Then, the tip was moved toward the substrate using the z-piezo stage over ~25-μm distance with a relatively high approach velocity (e.g., 0.5 μm/s) in the bulk solution, and then a slower velocity (e.g., 100 nm/s) was employed in the SECM feedback range to obtain the approach curve and determine substrate *z* position. All experiments were carried out at room temperature inside a Faraday cage.

In SECM feedback mode approach (*i*_T_ versus *d*) curve experiment, the tip and substrate currents can both be recorded as a function of the distance between the tip and the substrate. When Fc was used as the redox mediator, the tip was biased at a sufficiently positive potential to oxidize Fc at a rate governed by its diffusion (diffusion-limited current, *i*_T,∞_, is reached in this case). When the distance between the tip and the substrate (*d*) was sufficiently small (i.e., comparable to tip radius, *a*), Fc^+^ produced at the tip was reduced back to Fc at the conductive substrate (here RuNi/NC or NC) surface, causing an increase in the tip current with decreasing *d*. This is called SECM positive feedback; *i*_T_ > *i*_T,∞_. Otherwise, if the regeneration rate of Fc at the substrate was slow, *i*_T_ decreased with decreasing *d* because of the hindered diffusion of Fc, resulting in the SECM negative feedback; *i*_T_ < *i*_T,∞_.

When H^+^ acted as the redox mediator and the source of H_2_ in acidic solution, the tip was biased at a negative potential, *E*_T_ = −0.7 V versus Ag/AgCl, to reduce H^+^ (hydrogen evolution reaction) at its surface and the substrate was biased at +0.4 V versus Ag/AgCl for HOR. The solution contained 10 mM H_2_SO_4_ and 0.2 M K_2_SO_4_. When H_2_ came from H_2_O/OH^−^ reduction (H_2_O or OH^−^ or both) at the tip in basic (pH 10) solution, *E*_T_ was biased at −1.2 V versus Ag/AgCl. In both cases, negative feedback approach curves were obtained when the substrate was unbiased or not reactive toward HOR. SECM feedback mode images (fig. S14, C to F) were obtained by scanning the tip laterally (in the *x*-*y* plane) over the sample surface at a distance comparable to tip radius.

In the TG-SC mode of SECM imaging, (Fig. 4A) H_2_ was generated at the tip by H_2_O/OH^−^ reduction (H_2_O or OH^−^ or both) and oxidized at the substrate (here RuNi/NC). The potentials of the tip and substrate were −1.2 and +0.8 V versus Ag/AgCl, respectively. Before scanning in TG-SC mode, the tip was first brought close to the unbiased substrate using negative SECM feedback (fig. S13).

### In situ XAFS measurement

The in situ experiments were conducted at room temperature in a flow half-cell with H_2_-saturated 0.1 M KOH electrolyte. The sample was loaded on a glassy carbon electrode and immersed in the electrolyte. The Ru and Ni K-edge XAFS spectra were recorded at open circuit potential (0), 0.05, 0.1, 0.15, and 0.25 V versus RHE in the fluorescence mode at Beamline TPS 44A of the National Synchrotron Radiation Research Center.

### DFT calculations

Spin-polarized first principles calculations based on DFT (*45*, *46*) were performed using the projector-augmented wave method (*47*, *48*) as implemented in the Vienna ab initio simulation package (*48*). Electron exchange and correlation terms were described by semi-empirical Bayesian error estimation functional with van der Waals functional (*49*) that combines generalized gradient approximation with long-range dispersion correction derived from the DFT-D2 method of Grimme (*50*). The SACs and DASCs were modeled in a 6 × 6 graphene nanosheet, e.g., Ru/NC, Ni/NC, and RuNi/NC were modeled by RuN_4_, NiN_4_, and RuN_3_NiN_3_ centers, respectively. For all the calculations, cutoff energy of 500 eV was set and the Brillouin zone was sampled by gamma-centered 2 × 2 × 1 *k*-points generated by the Monkhorst-Pack scheme. The structural relaxations were converged when the residual force on each ion fell below 0.01 eV Å^−1^, and the convergence of electronic structure was reached when the energy difference between the two iterations was smaller than 10^−5^ eV per atom. We used a Fermi-level smearing width of 0.05 eV for the calculations of adsorbates, whereas 0.01 eV for nonadsorbed species, to improve the convergence. TS were located using the climbing image nudged elastic band method with the presence of potassium ion and water molecules (*51*, *52*).

### Gibbs free energy corrections for adsorbates and nonadsorbed species

The Gibbs free energy of *H and *OH binding (ΔG_*H_ and ΔG_*OH_) were determined by the computational hydrogen electrode model (*53*), where the Gibbs free energy for a proton/electron pair [*G*(H^+^ + e^−^)] in the electrolyte is treated equally to half of the Gibbs free energy of molecular H_2_, 0.5*G*(H_2_). *G* was calculated as *G* = *E* + ZPE + ∫*C*_p_d*T* − *TS*, where *E* is the electronic energy obtained from ab initio DFT calculations, ZPE, ∫*C*_p_d*T*, and *TS* are the contributions from zero-point energies, temperature enthalpic, and entropic corrections (*T* = 300 K), respectively.

## Supplementary Materials

### This PDF file includes:

Tables S1 to S4 Figs. S1 to S15 References
