# Supplementary material for: Design of Ru-Ni diatomic sites for efficient alkaline hydrogen oxidation
Source: Sci Adv. 2022 Jun 1;8(22):eabm3779. doi: 10.1126/sciadv.abm3779 (PMC9159574; doi:10.1126/sciadv.abm3779)
Supplement: Supplementary file 1 — Tables S1 to S4 Figs. S1 to S15 References [file sciadv.abm3779_sm.pdf]

Science Advances
AAAS

Supplementary Materials for

Design of Ru-Ni diatomic sites for efficient alkaline hydrogen oxidation

Lili Han *et al.*

Corresponding author: Xijun Liu, xjliu@tjut.edu.cn; Huolin L. Xin, huolin.xin@uci.edu

Sci. Adv. **8**, eabm3779 (2022)
DOI: 10.1126/sciadv.abm3779

This PDF file includes:

Tables S1 to S4
Figs. S1 to S15
References

Supplementary Figures and Tables

Table S1. Gibbs free energies of *H and *OH binding on different single-atom catalysts (SACs) and diatomic site catalysts (DASCs) from BEEF-vdW calculations.

SACs	ΔG_{*H}	ΔG_{*OH}	DASCs	ΔG_{*H}	ΔG_{*OH}
Ir	−0.23	0.94	Ir-Ni	−0.53	0.45
Rh	−0.06	0.92	Ir-Pd	−0.67	0.13
Ru	−0.32	0.02	Ir-Pt	−0.65	0.23
Ni	1.79	1.99	Rh-Ni	−0.31	0.51
Pd	1.96	2.29	Rh-Pd	−0.45	0.32
Pt	1.63	2.39	Rh-Pt	−0.61	0.32
			Ru-Ni	−0.23	−0.01
			Ru-Pd	−0.38	−0.38
			Ru-Pt	−0.47	−0.45

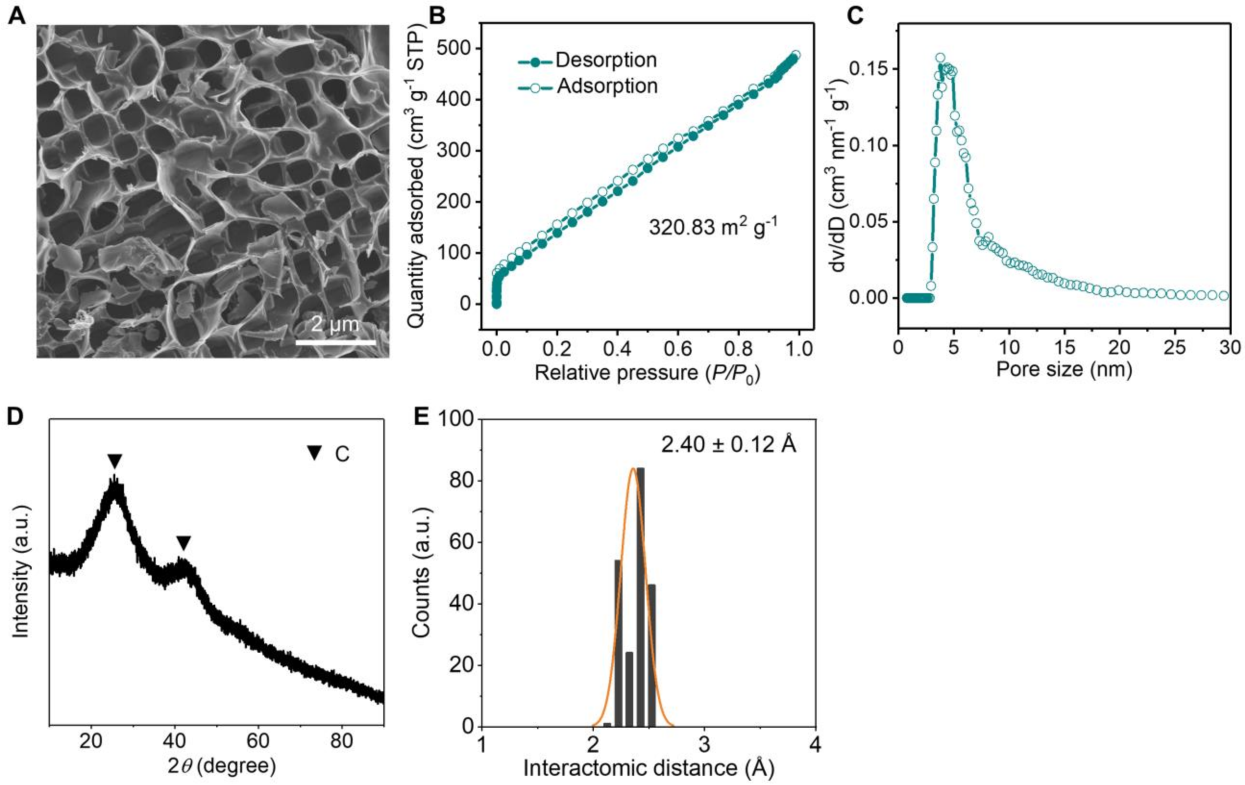

Fig. S1. Additional characterizations of RuNi/NC. (**A**) SEM image. (**B**) Nitrogen adsorption-desorption isotherm and (**C**) pore size distribution of RuNi/NC. The specific area was measured to be 320.68 m^2 g^{-1}. (**D**) XRD pattern. (**E**) Statistical interatomic distances of the observed diatomic pairs in atomic-resolution HAADF-STEM images of RuNi/NC.

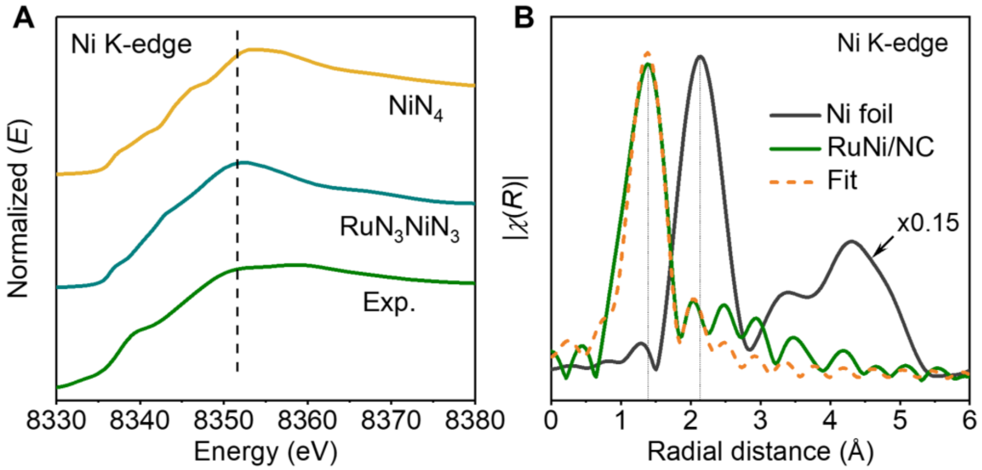

Fig. S2. Fitting of Ni K-edge XANES and EXAFS spectra for RuNi/NC. (**A**) Comparison between the experimental Ni K-edge XANES spectrum of RuNi/NC and the theoretical ones calculated based on the models of RuN_3NiN_3/graphene and NiN_4/graphene, which were generated after energy optimization. (**B**) Ni-K edge FT-EXAFS spectra of Ni foil, RuNi/NC, and the fit with the RuN_3NiN_3/graphene model.

Table S2. Structural parameters extracted from the EXAFS fittings for the RuNi/NC sample.

Element	Structure	Bonding	*R* (Å)	σ^2	*CN*	ΔE_0 (eV)	S_0^2	R-factor
Ru	NiN_3RuN_3	Ru-Ni	2.32±0.20	0.013	1.02±0.182	0.938	0.863	0.05
		Ru-N	1.93±0.10	0.002	2.87±0.35			
Ni	NiN_3RuN_3	Ni-Ru	2.32±0.10	0.006	0.96±0.66	−1.91	0.849	0.039
		Ni-N	1.89±0.04	0.019	2.46±0.72			
Ru	$Ru(Cl)N_3NiN_3$	Ru-Cl	2.30±0.06	0.0071	0.045±0.4	−0.98	0.865	0.043
Ru	$Ru(Cl)N_3Ni(Cl)N_3$	Ru-Cl	2.28±0.07	0.0068	0.09±0.6	−0.49	0.842	0.044

Note: *R*, the distance between absorber and backscatter atoms; σ^2, Debye–Waller factor; ΔE_0, edge-energy shifts; S_0^2, amplitude reduction factor; *R*-factor represents the goodness of fit. The pretreatment of data was performed using Athena and the oscillation in the k range from 2.5 ~ 8 $Å^{-1}$ was selected for further EXAFS fitting. The EXAFS fittings of the Ru and Ni K-edge spectra were performed using Artemis software.

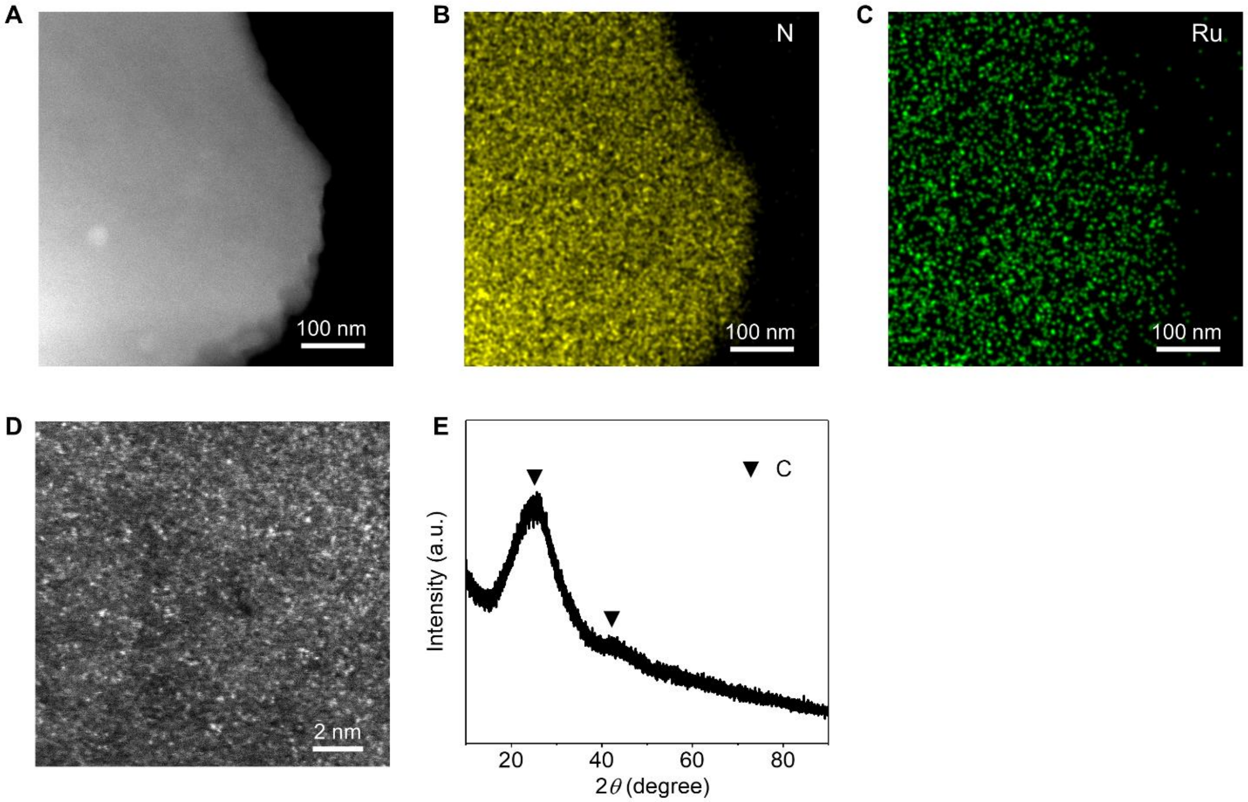

Fig. S3. Structural characterizations of Ru/NC. (**A−C**) HAADF-STEM image and corresponding STEM-EDS elemental maps of N and Ru. (**D**) Atomic-resolution HAADF-STEM image. (**E**) XRD pattern.

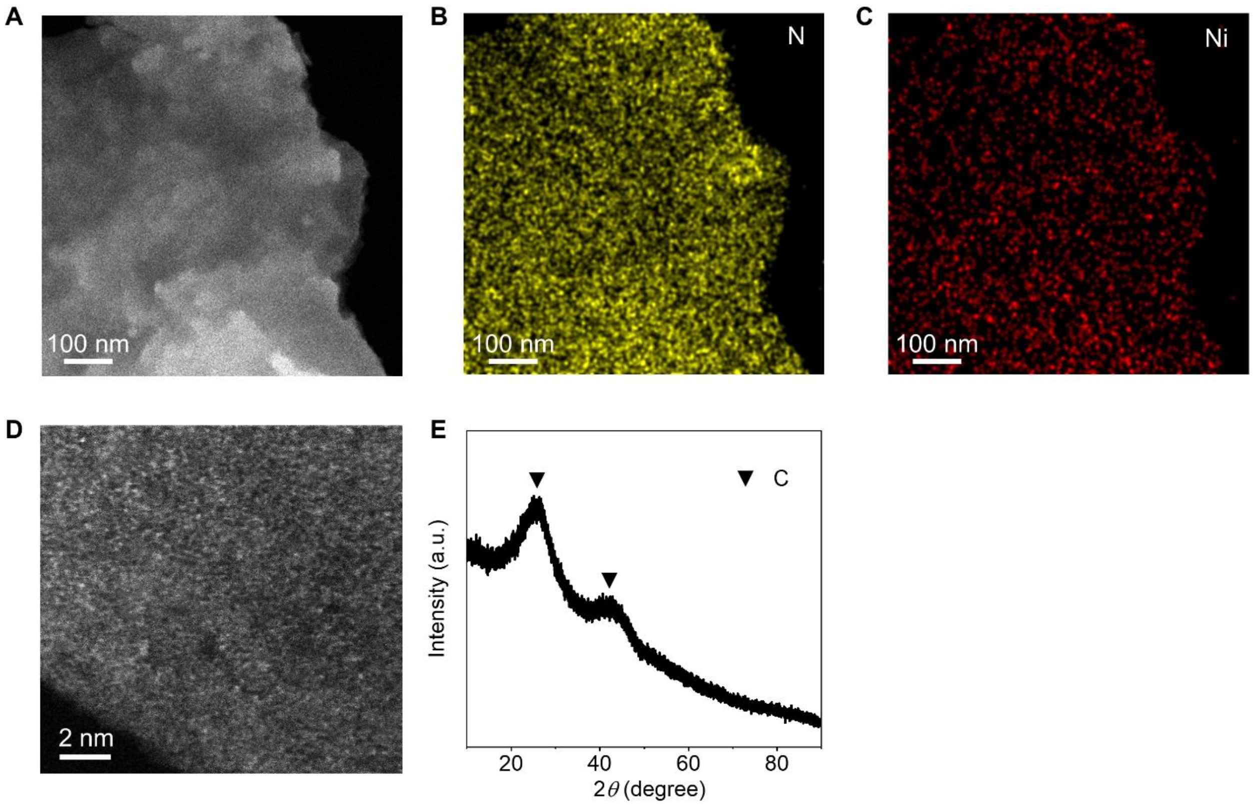

Fig. S4. Structural characterizations of Ni/NC. (**A−C**) HAADF-STEM image and corresponding STEM-EDS elemental maps of N and Ni. (**D**) Atomic-resolution HAADF-STEM image. (**E**) XRD pattern.

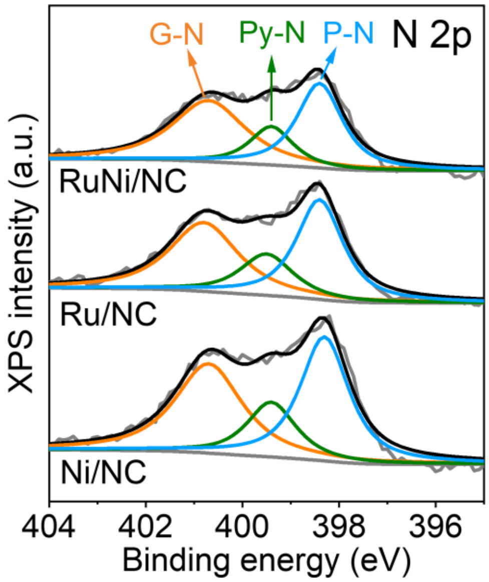

Fig. S5. N 2p XPS spectra of RuNi/NC, Ru/NC, and Ni/NC. P-N, Py-N, and G-N denote pyridinic N, pyrrolic N, and graphitic N, respectively. The nitrogen-doped carbon support contains pyridinic N, pyrrolic N, and graphitic N. Their content for each type of nitrogen in RuNi/NC, Ru/NC, and Ni/NC catalysts is similar to each other (table S3).

Table S3. Content of pyridinic N, pyrrolic N, and graphitic N in RuNi/NC, Ru/NC, and Ni/NC catalysts obtained from XPS measurements.

Catalysts	Pyridinic N at.%	Pyrrolic N at.%	Graphitic N at.%
RuNi/NC	4.54	1.69	3.92
Ru/NC	4.30	1.99	4.24
Ni/NC	4.12	1.86	4.17

Table S4. Comparison between electrocatalysts for alkaline HOR in prior studies and our work.

Electrocatalysts	$j_{g@0.05\ V}$ (vs. RHE)	$j_{k@0.05V}$ (vs. RHE)	$j_{m,k@0.05V}$ (vs. RHE)	j_0 (mA cm^{-2})	Stability	Reference
Ni/N-CNT	–	2.33 mA cm^{-2}	9.3 mA mg_{Ni}^{-1}	–	–	(23)
NiNPs	–	0.0024 mA cm^{-2}	0.28 mA mg_{Ni}^{-1}	–	–	(23)
Ni/CNT	–	0.018 mA cm^{-2}	1.9 mA mg_{Ni}^{-1}	–	–	(23)
Ni/N−CNT	–	0.075 mA cm^{-2}	9.3 mA mg_{Ni}^{-1}	–	–	(23)
np-Ni_3N	1.7 mA cm^{-2}	4.76 mA cm^{-2}	29.8 mA mg_{Ni}^{-1}	–	> 10,000 s	(54)
$Co_{0.17}Ni_{4.49}Mo_1$	–	0.044±0.005 mA cm^{-2}	–	0.015		(55)
$CeO_{2(r)}$-Ni/C-1	–	–	12.28 mA $mg_{cat.}^{-1}$	–	–	(54)
Ni/NiO/C-700	–	1.59 mA cm^{-2}	5.0 mA mg_{Ni}^{-1}	–	12 h	(56)
Ni-H_2-2%	–	–	50.4 mA $mg_{cat.}^{-1}$	–	–	(57)
V-Ni_3N/Ni	1.54 mA cm^{-2}	–	–	–	–	(58)
Ni/MoO_2	–	–	38.5 mA mg_{Ni}^{-1}	–	–	(59)
Ni-NiO_x/XC-72	–	0.083±0.001 mA cm^{-2}	32.1±4.8 mA mg_{Ni}^{-1}	0.056±0.0010	–	(60)
Ni_3N/C	–	3.9 mA cm^{-2}	24.38 mA mg_{Ni}^{-1}	–	5,000 CV	(61)
Ni/XC-72	–	–	5.89±0.71 mA mg_{Ni}^{-1}	0.016±0.002	–	(62)
$CeO_{2(r)}$-Ni/XC-72	–	–	1.28±0.27 mA mg_{Ni}^{-1}	0.038±0.0022	–	(62)
$CeO_{2(r)}$-Ni/C-1	–	1.73 mA cm^{-2}	12.28 mA mg_{Ni}^{-1}	–	1,000 CV	(62)
Ru NP/PC	1.04 mA cm^{-2} @ 0.02 V	–	–	–	1,000 CV	(63)
Ru/C (3.1 nm)	–	–	–	0.063	–	(64)
Pt/Cu NWs	–	10.9 mA cm^{-2}	–	2.1	–	(65)
Ir/C-800C	–	4.1 mA cm^{-2}	–	–	–	(66)
IrNi@Ir/C	–	–	1.12 mA mg_{Ir}^{-1}	–	1,000 CV	(15)
Ni@C-500°C	–	–	–	0.032	120 h	(67)
0.38CeO_x-Pd/C	–	–	–	0.118	24 h	(68)
55%Ni/SC	–	–	11 mA mg_{Ni}^{-1}	–	–	(69)
Ni/SC	–	–	8.6 mA $mg_{cat.}^{-1}$	–	–	(69)
Ir/CeO_2–C	–	–	73.5 mA mg_{Ir}^{-1}	–	2,000 CV	(70)
Pd/Cu NWs	–		–	1.01	–	(71)
Ru_3Ir_2/C	–	–	–	0.85	–	(72)
Pd-CeO_2/C	–	–	–	0.13	3,000 CV	(73)
Pd-CN_x	–	–	–	0.037	–	(74)
Pd/C-CeO_2	–	–	–	0.0545	–	(75)
Pd/C	0.084 mA cm^{-2}	–	–	–	–	(76)
$Ru_{0.80}Pt_{0.20}$	0.472 mA cm^{-2}	–	–	–	–	(76)

RuNi/NC	1.93±0.08 mA cm^{-2}	5.69±0.17 mA cm^{-2}	132.6±3.3 mA mg_{RuNi}^{-1}	2.69±0.06	30 h<br>5,000 CV	
Ru/NC	1.71±0.02 mA cm^{-2}	3.63±0.08 mA cm^{-2}	62.4±2.5 mA mg_{Ru}^{-1}	1.69±0.03	−	**This Work**
Ni/NC	0.99±0.03 mA cm^{-2}	1.62±0.04 mA cm^{-2}	38.5±1.3 mA mg_{Ni}^{-1}	0.95±0.04	−	

$j_{g@0.05\ V}$: Current per cm^2 (with respect to the geometric area of the electrode) obtained at 0.05 V.
$j_{k@0.05\ V}$: Kinetics current per cm^2 (with respect to the geometric area of the electrode) obtained at 0.05 V.
$j_{m,k@0.05V}$: Kinetics current per mg of metal obtained at 0.05 V.
j_0: Exchange current per cm^2 (with respect to the geometric area of the electrode).

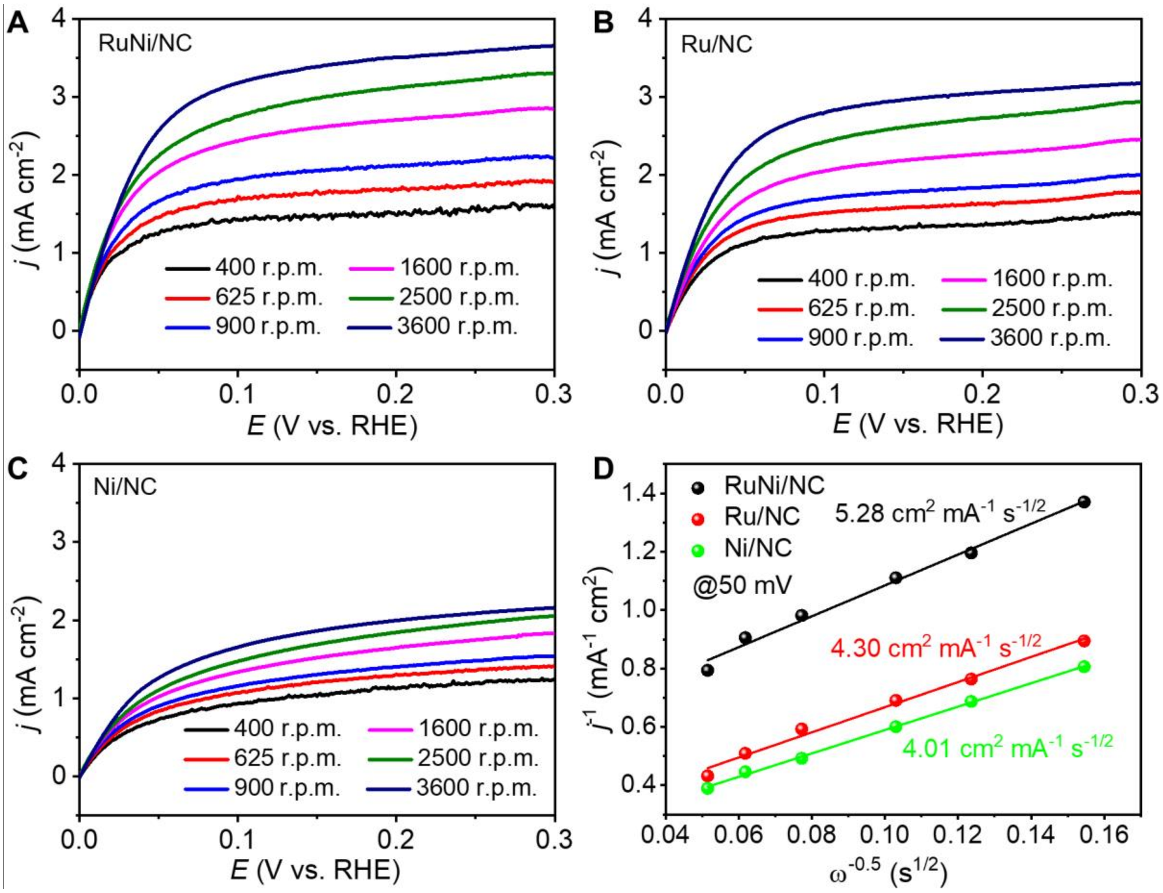

Fig. S6. HOR polarization curves of (**A**) RuNi/NC, (**B**) Ru/NC and (**C**) Ni/NC at various rotating speeds. (**D**) Koutecky–Levich plots at an overpotential of 50 mV.

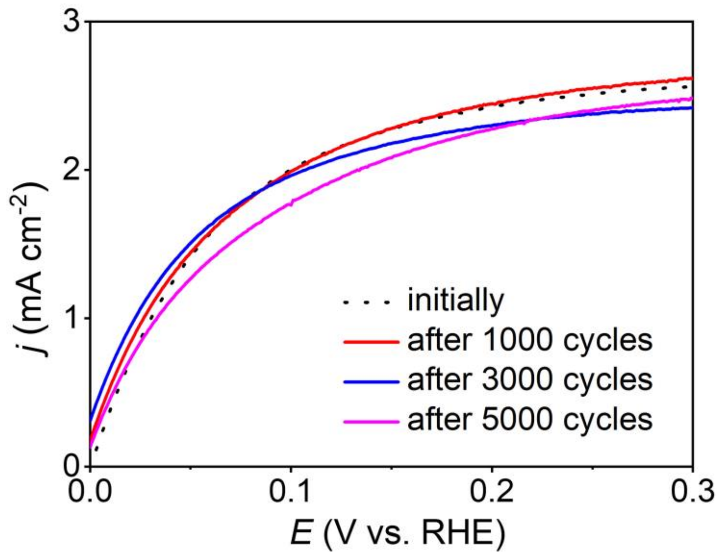

Fig. S7. Polarization curves of RuNi/NC in H_2/10 ppm CO-saturated 0.1 M KOH solution at a scan rate of 1 mV s^{-1} and rotating speed of 1,600 rpm. The current density decreases slightly by 3.1% after 5,000 cycles, suggesting good stability of the RuNi/NC catalyst.

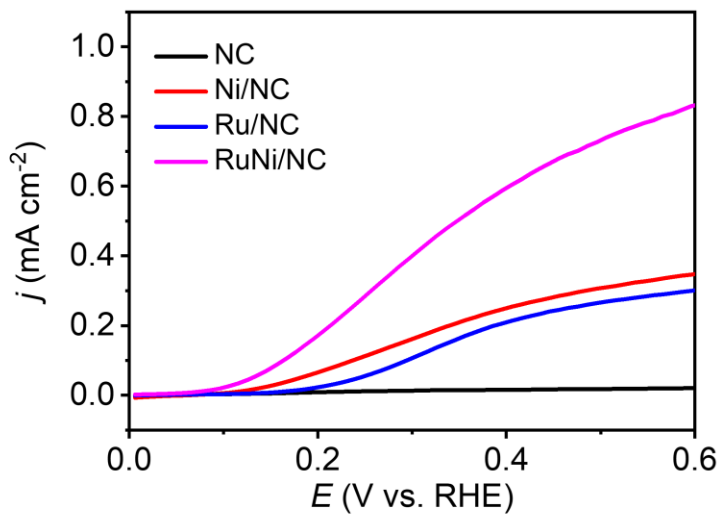

Fig. S8. Current density dependent on applied potential in CO-saturated 0.1 M KOH for RuNi/NC, Ru/NC, Ni/NC, and NC catalysts. We performed the electrochemical experiments on NC support, Ni/NC, Ru/NC, and RuNi/NC in CO-saturated 0.1 M KOH to explore the impacts of the factors on the CO poisoning resistance (*77*). Clearly, RuNi/NC has much better CO poisoning resistance than that of NC support, Ni/NC and Ru/NC, suggesting that the unique structure of Ru-Ni diatomic sites (their model is RuN_3NiN_3) plays a key role in anti-poisoning towards CO.

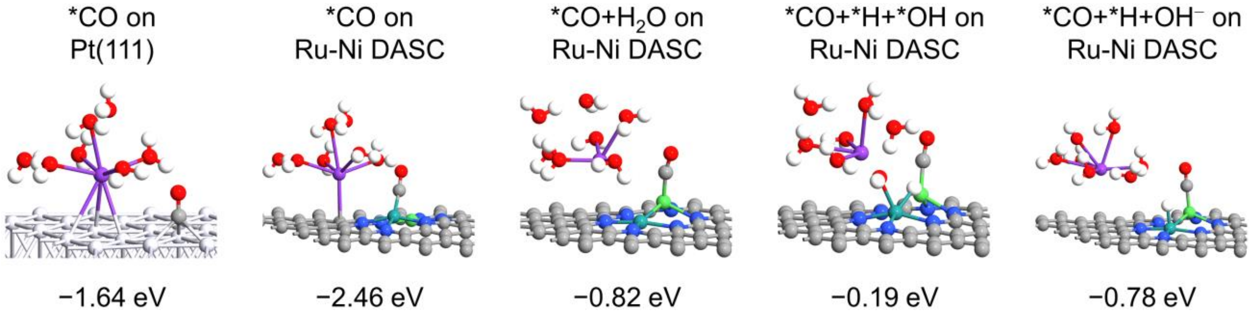

Fig. S9. DFT determined *CO adsorption energy on Pt(111) and Ru-Ni DASC without and with the presence of reaction intermediates of HOR.

Note: We performed DFT calculations to determine the *CO adsorption energy on Ru-Ni DASC for RuNi/NC and Pt(111) for Pt/C. If we do not consider the presence of reaction intermediates in HOR, the *CO adsorption on Ru-Ni DASC is stronger than that of Pt(111) (−2.46 vs. −1.64 eV), which suggests *CO is more readily adsorbed on the bridge site of Ru-Ni; in contrast, *CO adsorbs on the hollow site of Pt(111). However, when competing with different reaction intermediates of H_2O, *H+*OH, or *H+OH^- (Fig. 5A), we found that the *CO adsorption is much weakened on Ru-Ni DASC, can be more than 2 eV, due to the strong adsorbate-adsorbate interaction. On Ru-Ni DASC, all these reaction intermediates can be adsorbed on Ru and Ni atoms simultaneously, and during the HOR process, adsorbed *CO will easily desorb from these sites and avoid CO poisoning. On the other hand, *CO adsorption energy remains intact on Pt(111) since it offers different active sites for *CO adsorption and HOR. We expect the *CO will continuously adsorb on Pt(111) surface and increase the local *CO coverages and thus affect the thermodynamics and kinetics of HOR, and eventually, results in some CO poisoning effect. Thus, RuNi/NC has a higher resistance to CO poisoning than Pt/C. This is consistent with the electrochemical results in Fig. 3f and Fig. S8.

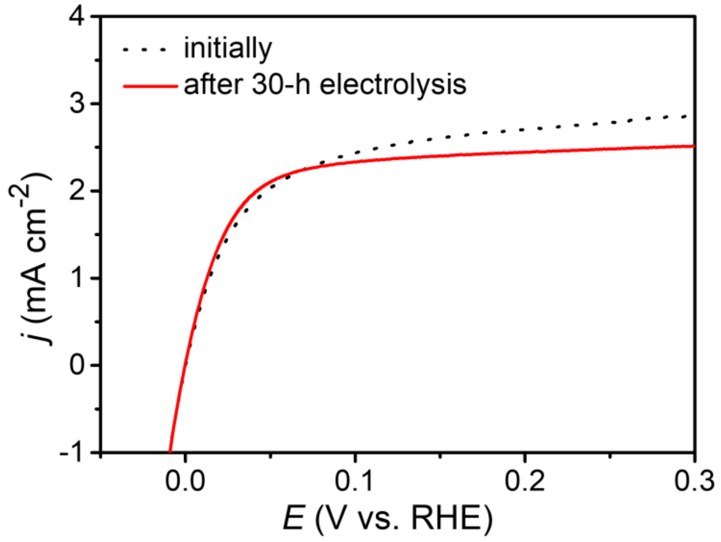

Fig. S10. HOR polarization curves of RuNi/NC before and after the 30-h stability test.

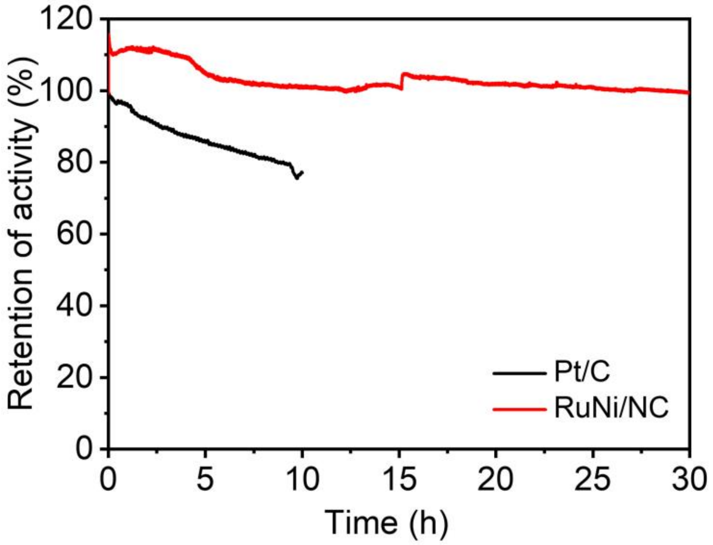

Fig. S11. **Activity retention of the current relative to the initial current vs. electrolysis time for RuNi/NC and Pt/C.**

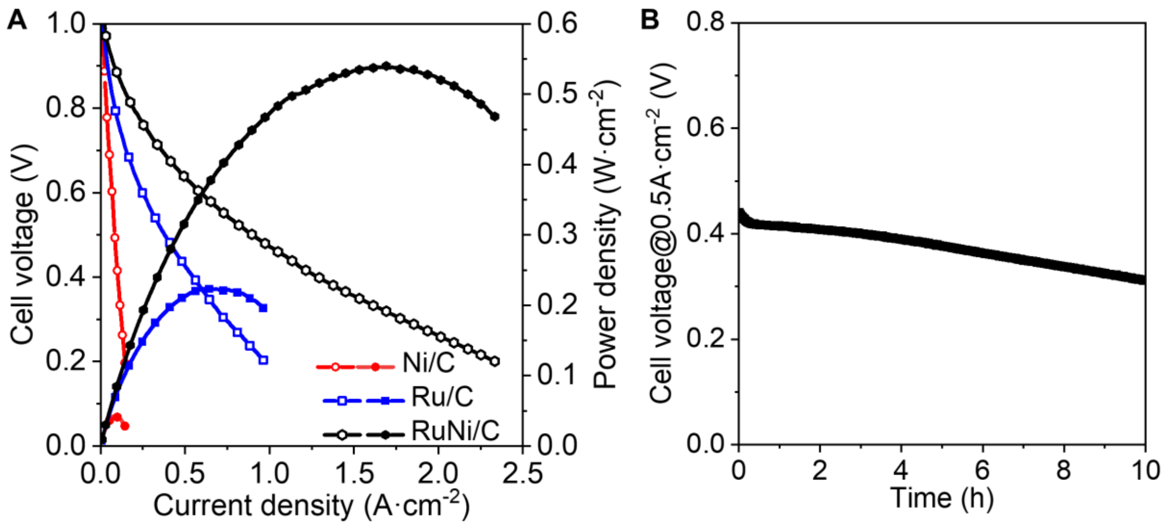

Fig. S12. Fuel cell measurements. (**A**) AEMFC cell performances of RuNi/NC, Ru/C, and Ni/NC. (**B**) Voltage degradation vs. time of RuNi/NC at current density of 0.5 A cm^{-2}.

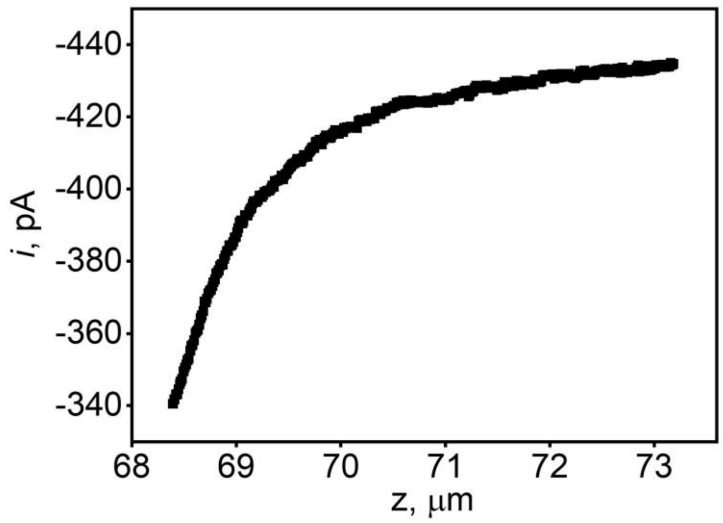

Fig. S13. SECM approach curve recorded at the tip electrode in 0.1 M PB (pH = 10). Substrate unbiased, E_{tip}= −1.2V vs. Ag/AgCl.

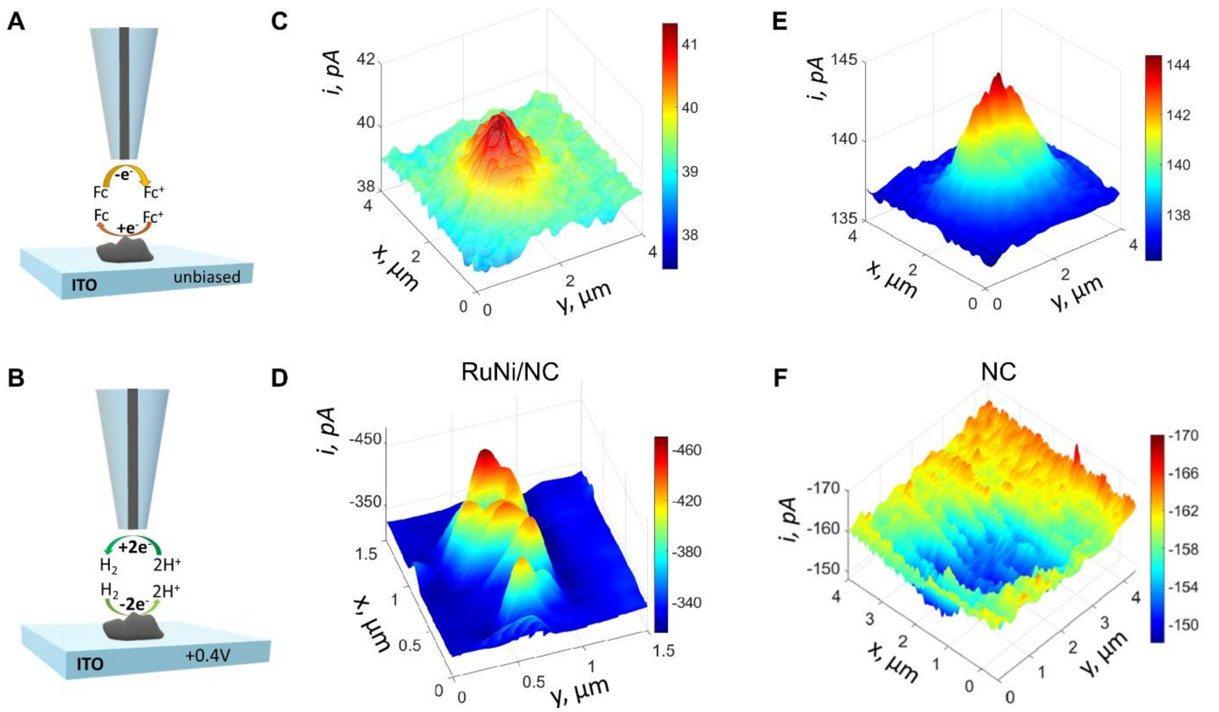

Fig. S14. Mapping topography and HOR activity of RuNi/NC and NC catalysts on the ITO surface with SECM. Schematic representation of imaging topography/conductivity (**A**) and electrocatalytic activity for HOR (**B**) by feedback mode SECM experiments. Feedback mode SECM images in Fc (**C, E**) and H_2SO_4 (**D, F**) of RuNi/NC (**C, D**) and NC (**E, F**) samples. Solution contains (**C, E**) 1 mM Fc and 0.1 M KCl, E_T = +0.4 V vs. Ag/AgCl, the substrate is unbiased. (**D, F**) 10 mM H_2SO_4, and 0.2 M K_2SO_4, E_T = −0.7 V vs. Ag/AgCl, E_S = +0.4 V vs. Ag/AgCl.

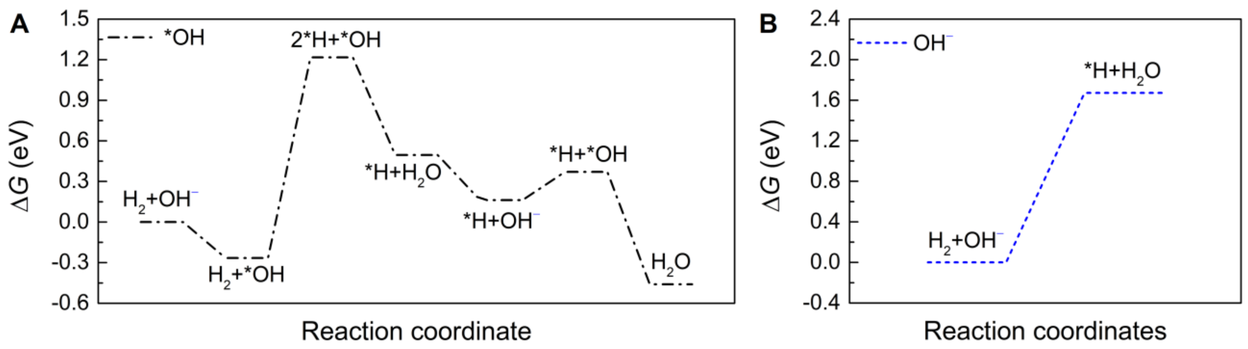

Fig. S15. HOR mechanism on Ru and Ni SACs. Free energy diagram of HOR on (**A**) Ru and (**B**) Ni SACs obtained from DFT calculations.

Note: Our DFT results suggest that the H_2 dissociation on Ru-Ni DASC can be achieved in both Tafel and Heyrovsky steps. The kinetics of H_2O formation is much faster than on Ru and Ni SACs since the Tafel pathway only involves the desorption of reaction intermediates. In contrast, the Tafel step of H_2 dissociation on Ru SAC is more energetically unfavorable due to a relatively large ΔG for the potential-determining step (fig. S15A), which suggests a decrease in the reaction rate of HOR. Speaking of Ni SAC, the H_2 dissociation can only happen with the help of OH^- and simultaneously transform one of the H atoms into H_2O (Heyrovsky step) with an even larger ΔG, and only one H in H_2 molecules undergoes the HOR to produce H_2O, which indicates more sluggish kinetics of HOR.

REFERENCES AND NOTES

1. B. P. Setzler, Z. Zhuang, J. A. Wittkopf, Y. Yan, Activity targets for nanostructured platinum-group-metal-free catalysts in hydroxide exchange membrane fuel cells. *Nat. Nanotechnol.* **11**, 1020–1025 (2016).

2. Y. Xue, L. Shi, X. Liu, J. Fang, X. Wang, B. P. Setzler, W. Zhu, Y. Yan, Z. Zhuang, A highly-active, stable and low-cost platinum-free anode catalyst based on RuNi for hydroxide exchange membrane fuel cells. *Nat. Commun.* **11**, 5651 (2020).

3. J. Durst, C. Simon, F. Hasche, H. A. Gasteiger, Hydrogen oxidation and evolution reaction kinetics on carbon supported Pt, Ir, Rh, and Pd electrocatalysts in acidic media. *J. Electrochem. Soc.* **162**, F190–F203 (2015).

4. L. An, X. Zhao, T. Zhao, D. Wang, Atomic-level insight into reasonable design of metal-based catalysts for hydrogen oxidation in alkaline electrolytes. *Energ. Environ. Sci.* **14**, 2620–2638 (2021).

5. L. Su, D. Gong, Y. Jin, D. Wu, W. Luo, Recent advances in alkaline hydrogen oxidation reaction. *J. Energ. Chem.* **66**, 107–122 (2022).

6. J. Durst, A. Siebel, C. Simon, F. Hasché, J. Herranz, H. A. Gasteiger, New insights into the electrochemical hydrogen oxidation and evolution reaction mechanism. *Energ. Environ. Sci.* **7**, 2255–2260 (2014).

7. H. A. Miller, A. Lavacchi, F. Vizza, M. Marelli, F. Di Benedetto, F. D'Acapito, Y. Paska, M. Page, D. R. Dekel, A Pd/C-CeO_2 anode catalyst for high-performance platinum-free anion exchange membrane fuel cells. *Angew. Chem. Int. Ed.* **128**, 6108–6111 (2016).

8. V. Briega-Martos, A. Ferre-Vilaplana, E. Herrero, J. M. Feliu, Why the activity of the hydrogen oxidation reaction on platinum decreases as pH increases. *Electrochim. Acta* **354**, 136620 (2020).

9. H. Wang, H. D. Abruña, Rh and Rh alloy nanoparticles as highly active H_2 oxidation catalysts for alkaline fuel cells. *ACS Catal.* **9**, 5057–5062 (2019).

10. Y. Wang, G. Wang, G. Li, B. Huang, J. Pan, Q. Liu, J. Han, L. Xiao, J. Lu, L. Zhuang, Pt-Ru catalyzed hydrogen oxidation in alkaline media: Oxophilic effect or electronic effect? *Energ. Environ. Sci.* **8**, 177–181 (2014).

11. S. Lu, Z. Zhuang, Investigating the influences of the adsorbed species on catalytic activity for hydrogen oxidation reaction in alkaline electrolyte. *J. Am. Chem. Soc.* **139**, 5156–5163 (2017).

12. G. Wang, W. Li, B. Huang, L. Xiao, J. Lu, L. Zhuang, Exploring the composition-activity relation of Ni-Cu binary alloy electrocatalysts for hydrogen oxidation reaction in alkaline media. *ACS Appl. Energ. Mater.* **2**, 3160–3165 (2019).

13. B. Qin, H. Yu, J. Jia, C. Jun, X. Gao, D. Yao, X. Sun, W. Song, B. Yi, Z. Shao, A novel IrNi@PdIr/C core-shell electrocatalyst with enhanced activity and durability for the hydrogen oxidation reaction in alkaline anion exchange membrane fuel cells. *Nanoscale* **10**, 4872–4881 (2018).

14. E. Liu, L. Jiao, J. Li, T. Stracensky, Q. Sun, S. Mukerjee, Q. Jia, Interfacial water shuffling the intermediates of hydrogen oxidation and evolution reactions in aqueous media. *Energ. Environ. Sci.* **13**, 3064–3074 (2020).

15. D. Liu, S. Lu, Y. Xue, Z. Guan, J. Fang, W. Zhu, Z. Zhuang, One-pot synthesis of IrNi@Ir core-shell nanoparticles as highly active hydrogen oxidation reaction electrocatalyst in alkaline electrolyte. *Nano Energy* **59**, 26–32 (2019).

16. Y. Duan, Z.-Y. Yu, L. Yang, L.-R. Zheng, C.-T. Zhang, X.-T. Yang, F.-Y. Gao, X.-L. Zhang, X. Yu, R. Liu, H.-H. Ding, C. Gu, X.-S. Zheng, L. Shi, J. Jiang, J.-F. Zhu, M.-R. Gao, S.-H. Yu, Bimetallic nickel-molybdenum/tungsten nanoalloys for high-efficiency hydrogen oxidation catalysis in alkaline electrolytes. *Nat. Commun.* **11**, 4789 (2020).

17. G. Huang, W. Liang, Y. Wu, J. Li, Y. Jin, H. Zeng, H. Zhang, F. Xie, J. Chen, N. Wang, Y. Jin, H. Meng, Co_2P/CoP hybrid as a reversible electrocatalyst for hydrogen oxidation/evolution reactions in alkaline medium. *J. Catal.* **390**, 23–29 (2020).

18. C.-P. Huang, M.-C. Tsai, X.-M. Wang, H.-S. Cheng, Y. -H. Mao, C. -J. Pan, J. -N. Lin, L. -D. Tsai, T. -S. Chan, W. -N. Su, B. -J. Hwang, Engineering heterometallic bonding in bimetallic electrocatalysts: Towards optimized hydrogen oxidation and evolution reactions. *Cat. Sci. Technol.* **10**, 893–903 (2020).

19. Y. Qiu, L. Xin, Y. Li, I. T. McCrum, F. Guo, T. Ma, Y. Ren, Q. Liu, L. Zhou, S. Gu, M. J. Janik, W. Li, BCC-phased PdCu alloy as a highly active electrocatalyst for hydrogen oxidation in alkaline electrolytes. *J. Am. Chem. Soc.* **140**, 16580–16588 (2018).

20. J. Mao, C.-T. He, J. Pei, Y. Liu, J. Li, W. Chen, D. He, D. Wang, Y. Li, Isolated Ni atoms dispersed on Ru nanosheets: High-performance electrocatalysts toward hydrogen oxidation reaction. *Nano Lett.* **20**, 3442–3448 (2020).

21. J. Ohyama, T. Sato, Y. Yamamoto, S. Arai, A. Satsuma, Size specifically high activity of Ru nanoparticles for hydrogen oxidation reaction in alkaline electrolyte. *J. Am. Chem. Soc.* **135**, 8016–8021 (2013).

22. Y. Zhou, Z. Xie, J. Jiang, J. Wang, X. Song, Q. He, W. Ding, Z. Wei, Lattice-confined Ru clusters with high CO tolerance and activity for the hydrogen oxidation reaction. *Nat. Catal.* **3**, 454–462 (2020).

23. Z. Zhuang, S. A. Giles, J. Zheng, G. R. Jenness, S. Caratzoulas, D. G. Vlachos, Y. Yan, Nickel supported on nitrogen-doped carbon nanotubes as hydrogen oxidation reaction catalyst in alkaline electrolyte. *Nat. Commun.* **7**, 10141 (2016).

24. E. S. Davydova, F. D. Speck, M. T. Y. Paul, D. R. Dekel, S. Cherevko, Stability limits of Ni-based hydrogen oxidation electrocatalysts for anion exchange membrane fuel cells. *ACS Catal.* **9**, 6837–6845 (2019).

25. S. Ji, Y. Chen, X. Wang, Z. Zhang, D. Wang, Y. Li, Chemical synthesis of single atomic site catalysts. *Chem. Rev.* **14,** 2620–2638 (2020).

26. Y. Chen, S. Ji, C. Chen, Q. Peng, D. Wang, Y. Li, Single-atom catalysts: Synthetic strategies and electrochemical applications. *Joule* **2**, 1242–1264 (2018).

27. L. Han, M. Hou, P. Ou, H. Cheng, Z. Ren, Z. Liang, J. A. Boscoboinik, A. Hunt, I. Waluyo, S. Zhang, L. Zhuo, J. Song, X. Liu, J. Luo, H. L. Xin, Local modulation of single-atomic Mn sites for enhanced ambient ammonia electrosynthesis. *ACS Catal.* **11**, 509–516 (2020).

28. L. Han, X. Liu, J. Chen, R. Lin, H. Liu, F. Lü, S. Bak, Z. Liang, S. Zhao, E. Stavitski, J. Luo, R. R. Adzic, H. L. Xin, Atomically dispersed molybdenum catalysts for efficient ambient nitrogen fixation. *Angew. Chem. Int. Ed.* **58**, 2321–2325 (2019).

29. L. Han, Z. Ren, P. Ou, H. Cheng, N. Rui, L. Lin, X. Liu, L. Zhuo, J. Song, J. Sun, J. Luo, H. L. Xin, Modulating single-atom palladium sites with copper for enhanced ambient ammonia electrosynthesis. *Angew. Chem. Int. Ed.* **60**, 345–350 (2021).

30. L. Han, S. Song, M. Liu, S. Yao, Z. Liang, H. Cheng, Z. Ren, W. Liu, R. Lin, G. Qi, X. Liu, Q. Wu, J. Luo, H. L. Xin, Stable and efficient single-atom Zn catalyst for CO_2 reduction to CH_4. *J. Am. Chem. Soc.* **142**, 12563–12567 (2020).

31. Y. Han, Y. Wang, R. Xu. W. Chen, L. Zheng, A. Han, Y. Zhu, J. Zhang, H. Zhang, J. Luo, C. Chen, Q. Peng, D. Wang, Y. Li, Electronic structure engineering to boost oxygen reduction activity by controlling the coordination of the central metal. *Energ. Environ. Sci.* **11**, 2348–2352 (2018).

32. D. Strmcnik, M. Uchimura, C. Wang, R. Subbaraman, N. Danilovic, V. Dennis, A. P. Paulikas, V. R. Stamenkovic, N. M. Markovic, Improving the hydrogen oxidation reaction rate by promotion of hydroxyl adsorption. *Nat. Chem.* **5**, 300–306 (2013).

33. R. Subbaraman, D. Tripkovic, K.-C. Chang, D. Strmcnik, A. P. Paulikas, P. Hirunsit, M. Chan, J. Greeley, V. Stamenkovic, N. M. Markovic, Trends in activity for the water electrolyser reactions on 3d M(Ni,Co,Fe,Mn) hydr(oxy)oxide catalysts. *Nat. Mater.* **11**, 550–557 (2012).

34. I. T. McCrum, M. T. M. Koper, The role of adsorbed hydroxide in hydrogen evolution reaction kinetics on modified platinum. *Nat. Energy* **5**, 891–899 (2020).

35. W. Sheng, H. A. Gasteiger, Y. Shao-Horn, Hydrogen oxidation and evolution reaction kinetics on platinum: Acid vs alkaline electrolytes. *J. Electrochem. Soc.* **157,** B1529 (2010).

36. H. Sun, C.-W. Tung, Y. Qiu, W. Zhang, Q. Wang, Z. Li, J. Tang, H.-C. Chen, C. Wang, H. Chen, Atomic metal-support interaction enables reconstruction-free dual-site electrocatalyst. *J. Am. Chem. Soc.* **144**, 1174–1186 (2021).

37. J. Ohyama, T. Sato, A. Satsum, High performance of Ru nanoparticles supported on carbon for anode electrocatalyst of alkaline anion exchange membrane fuel cell. *J. Power Sources* **225**, 311–315 (2013).

38. S. Kabir, K. Lemire, K. Artyushkova, A. Roy, M. Odgaard, D. Schlueter, A. Oshchepkov, A. Bonnefont, E. Savinova, D. C. Sabarirajan, P. Mandal, E. J. Crumlin, I. V. Zenyuk, P. Atanassov, A. Serov, Platinum group metal-free NiMo hydrogen oxidation catalysts: High performance and durability in alkaline exchange membrane fuel cells. *J. Mater. Chem. A* **5**, 24433–24443 (2017).

39. T. Sun, H. Zhang, X. Wang, J. Liu, C. Xiao, S. U. Nanayakkara, J. L. Blackburn, M. V. Mirkin, E. M. Miller, Nanoscale mapping of hydrogen evolution on metallic and semiconducting MoS_2 nanosheets. *Nanoscale Horiz.* **4**, 619–624 (2019).

40. A. Djire, X. Wang, C. Xiao, O. C. Nwamba, M. V. Mirkin, N. R. Neale, Basal plane hydrogen evolution activity from mixed metal nitride MXenes measured by scanning electrochemical microscopy. *Adv. Funct. Mater.* **30**, 2001136 (2020).

41. T. Sun, D. Wang, M. V. Mirkin, H. Cheng, J.-C. Zheng, R. M. Richards, F. Lin, H. L. Xin, Direct high-resolution mapping of electrocatalytic activity of semi-two-dimensional catalysts with single-edge sensitivity. *Proc. Natl. Acad. Sci. U.S.A.* **116**, 11618–11623 (2019).

42. G. Han, Y. Zheng, X. Zhang, Z. Wang, Y. Gong, C. Du, M. N. Banis, Y.-M. Yiu, T.-K. Sham, L. Gu, Y. Sun, Y. Wang, J. Wang, Y. Gao, G. Yin, X. Sun, High loading single-atom Cu dispersed on graphene for efficient oxygen reduction reaction. *Nano Energy* **66**, 104088 (2019).

43. P. Sun, M. V. Mirkin, Kinetics of electron-transfer reactions at nanoelectrodes. *Anal. Chem.* **78**, 6526–6534 (2006).

44. Nioradze, R. Chen, J. Kim, M. Shen, P. Santhosh, S. Origins of nanoscale damage to glass-sealed platinum electrodes with submicrometer and nanometer size. *Anal. Chem.* **85**, 6198–6202 (2013).

45. W. Kohn, L. J. Sham, Self-consistent equations including exchange and correlation effects. *Phys. Rev.* **140**, A1133–A1138 (1965).

46. G. G. Kresse, J. J. Furthmüller, Efficient iterative schemes for ab initio total-energy calculations using a plane-wave basis set. *Phys. Rev. B* **54**, 11169–11186 (1996).

47. P. E. BlöChl, Projector augmented-wave method. *Phys. Rev. B* **50**, 17953–17979 (1994).

48. G. Kresse, D. Joubert, From ultrasoft pseudopotentials to the projector augmented-wave method. *Phys. Rev. B* **59**, 1758–1775 (1999).

49. J. Wellendorff, K. T. Lundgaard, A. Møgelhøj, V. Petzold, D. D. Landis, J. K. Nørskov, T. Bligaard, K. W. Jacobsen, Density functionals for surface science: Exchange-correlation model development with Bayesian error estimation. *Phys. Rev. B* **85**, 235149 (2012).

50. S. Grimme, Semiempirical GGA-type density functional constructed with a long-range dispersion correction. *J. Comput. Chem.* **27**, 1787–1799 (2006).

51. G. Henkelman, B. P. Uberuaga, H. Jonsson, A climbing image nudged elastic band method for finding saddle points and minimum energy paths. *J. Chem. Phys.* **113**, 9901–9904 (2000).

52. G. Henkelman, H. Jonsson, Improved tangent estimate in the nudged elastic band method for finding minimum energy paths and saddle points. *J. Chem. Phys.* **113**, 9978–9985 (2000).

53. J. K. Nørskov, J. Rossmeisl, A. Logadottir, L. Lindqvist, J. R. Kitchin, T. Bligaard, H. Jónsson, Origin of the overpotential for oxygen reduction at a fuel-cell cathode. *J. Phys. Chem. B* **108**, 17886–17892 (2004).

54. T. Wang, M. Wang, H. Yang, M. Xu, C. Zuo, K. Feng, M. Xie, J. Deng, J. Zhong, W. Zhou, T. Cheng, Y. Li, Correction: Weakening hydrogen adsorption on nickel via interstitial nitrogen doping promotes bifunctional hydrogen electrocatalysis in alkaline solution. *Energ. Environ. Sci.* **12**, 3611–3611 (2019).

55. W. Sheng, A. P. Bivens, M. Myint, Z. Zhuang, R. V. Forest, Q. Fang, J. G. Chen, Y. Yan, Non-precious metal electrocatalysts with high activity for hydrogen oxidation reaction in alkaline electrolytes. *Energ. Environ. Sci.* **7**, 1719–1724 (2014).

56. Y. Yang, X. Sun, G. Han, X. Liu, X. Zhang, Y. Sun, M. Zhang, Z. Cao, Y. Sun, Enhanced electrocatalytic hydrogen oxidation on Ni/NiO/C derived from a nickel-based metal-organic framework. *Angew. Chem. Int. Ed.* **58**, 10644–10649 (2019).

57. W. Ni, T. Wang, P. A. Schouwink, Y.-C. Chuang, H. M. Chen, X. Hu, Efficient hydrogen oxidation catalyzed by strain-engineered nickel nanoparticles. *Angew. Chem. Int. Ed.* **59**, 10797–10801 (2020).

58. H. Zhang, J. Wang, F. Qin, H. Liu, C. Wang, V-doped Ni_3N/Ni heterostructure with engineered interfaces as a bifunctional hydrogen electrocatalyst in alkaline solution: Simultaneously improving water dissociation and hydrogen adsorption. *Nano Res.* **14**, 3489–3496 (2021).

59. S. Deng, X. Liu, X. Guo, T. Zhao, Y. Lu, J. Cheng, K. Chen, T. Shen, Y. Zhu, D. Wang, Insight into the hydrogen oxidation electrocatalytic performance enhancement on Ni via oxophilic regulation of MoO_2. *J. Energ. Chem.* **54**, 202–207 (2021).

60. A. G. Oshchepkov, A. Bonnefont, S. N. Pronkin, O. V. Cherstiouk, C. Ulhaq-Bouillet, V. Papaefthimiou, V. N. Parmon, E. R. Savinova, Nanostructured nickel nanoparticles supported on vulcan carbon as a highly active catalyst for the hydrogen oxidation reaction in alkaline media. *J. Power Sources* **402**, 447–452 (2018).

61. W. Ni, A. Krammer, C.-S. Hsu, H. M. Chen, A. Schueler, X. Hu, Ni_3N as an active hydrogen oxidation reaction catalyst in alkaline medium. *Angew. Chem. Int. Ed.* **58**, 7445–7449 (2019).

62. F. Yang, X. Bao, P. Li, X. Wang, G. Cheng, S. Chen, W. Luo, Boosting hydrogen oxidation activity of Ni in alkaline media through oxygen-vacancy-rich CeO_2/Ni heterostructures. *Angew. Chem. Int. Ed.* **58**, 14179–14183 (2019).

63. M. Ming, Y. Zhang, C. He, L. Zhao, S. Niu, G. Fan, J. S. Hu, Room-temperature sustainable synthesis of selected platinum group metal (PGM = Ir, Rh, and Ru) nanocatalysts well-dispersed on porous carbon for efficient hydrogen evolution and oxidation. *Small* **15**, 1903057 (2019).

64. J. Zheng, W. Sheng, Z. Zhuang, B. Xu, Y. Yan, Universal dependence of hydrogen oxidation and evolution reaction activity of platinum-group metals on pH and hydrogen binding energy. *Sci. Adv.* **2**, e1501602 (2016).

65. S. M. Alia, B. S. Pivovar, Y. Yan, Platinum-coated copper nanowires with high activity for hydrogen oxidation reaction in base. *J. Am. Chem. Soc.* **135**, 13473–13478 (2013).

66. J. Zheng, Z. Zhuang, B. Xu, Y. Yan, Correlating hydrogen oxidation/evolution reaction activity with the minority weak hydrogen-binding sites on Ir/C catalysts. *ACS Catal.* **5**, 4449–4455 (2015).

67. Y. Gao, H. Peng, Y. Wang, G. Wang, L. Xiao, J. Lu, L. Zhuang, Improving the antioxidation capability of the Ni catalyst by carbon shell coating for alkaline hydrogen oxidation reaction. *ACS Appl. Mater. Interfaces* **12**, 31575–31581 (2020).

68. R. K. Singh, E. S. Davydova, J. Douglin, A. O. Godoy, H. Tan, M. Bellini, B. J. Allen, J. Jankovic, H. A. Miller, A. C. Alba-Rubio, Synthesis of CeO_x-decorated Pd/C catalysts by controlled surface reactions for hydrogen oxidation in anion exchange membrane fuel cells. *Adv. Funct. Mater.* **30**, 2002087 (2020).

69. F. Yang, X. Bao, Y. Zhao, X. Wang, G. Cheng, W. Luo, Enhanced HOR catalytic activity of PGM-free catalysts in alkaline media: The electronic effect induced by different heteroatom doped carbon supports. *J. Mater. Chem. A* **7**, 10936–10941 (2019).

70. B. Qin, H. Yu, J. Chi, J. Jia, X. Gao, D. Yao, B. Yi, Z. Shao, A novel Ir/CeO_2–C nanoparticle electrocatalyst for the hydrogen oxidation reaction of alkaline anion exchange membrane fuel cells. *RSC Adv.* **7**, 31574–31581 (2017).

71. S. M. Alia, Y. Yan, Palladium coated copper nanowires as a hydrogen oxidation electrocatalyst in base. *J. Electrochem. Soc.* **162**, F849 (2015), F853.

72. J. Ohyama, D. Kumada, A. Satsuma, Improved hydrogen oxidation reaction under alkaline conditions by ruthenium–iridium alloyed nanoparticles. *J. Mater. Chem. A* **4**, 15980–15985 (2016).

73. H. Yu, E. S. Davydova, U. Ash, H. A. Miller, L. Bonville, D. R. Dekel, R. Maric, Palladium-ceria nanocatalyst for hydrogen oxidation in alkaline media: Optimization of the Pd–CeO_2 interface. *Nano Energy* **57**, 820–826 (2019).

74. T. Bhowmik, M. K. Kundu, S. Barman, Palladium nanoparticle–graphitic carbon nitride porous synergistic catalyst for hydrogen evolution/oxidation reactions over a broad range of pH and correlation of its catalytic activity with measured hydrogen binding energy. *ACS Catal.* **6**, 1929–1941 (2016).

75. H. A. Miller, A. Lavacchi, F. Vizza, M. Marelli, F. Di Benedetto, F. D'Acapito, Y. Paska, M. Page, D. R. Dekel, A Pd/C-CeO_2 anode catalyst for high-performance platinum-free anion exchange membrane fuel cells. *Angew. Chem. Int. Ed.* **55**, 6004–6007 (2016).

76. S. St. John, R. W. Atkinson, R. R. Unocic, T. A. Zawodzinski Jr., A. B. Papandrew, Ruthenium-alloy electrocatalysts with tunable hydrogen oxidation kinetics in alkaline electrolyte. *J. Phys. Chem. C* **119**, 13481–13487 (2015).

77. X. Yang, Y. Wang, X. Wang, B. Mei, E. Luo, Y. Li, Q. Meng, Z. Jin, Z. Jiang, C. Liu, J. Ge, W. Xing, CO-tolerant PEMFC anodes enabled by synergistic catalysis between iridium single-atom sites and nanoparticles. *Angew. Chem. Int. Ed.* **60**, 26177–26183 (2021).
